# Supplementary material for: Endothelium-Independent Vasodilatory Effects of Isodillapiolglycol Isolated from Ostericum citriodorum
Source: Molecules. 2020 Feb 17;25(4):885. doi: 10.3390/molecules25040885 (PMC7070945; doi:10.3390/molecules25040885)
Supplement: Supplementary file 1 [file molecules-25-00885-s001.zip › English-editing-certificate-tengshuo luo.pdf]

We certify that the following article

# Endothelium-independent vasodilatory effects of isodillapiolglycol isolated from Ostericum citriodorum

tengshuo luo

has undergone English language editing by MDPI. The text has been checked for correct use of grammar and common technical terms, and edited to a level suitable for reporting research in a scholarly journal.
MDPI uses experienced, native English speaking editors. Full details of the editing service can be found at
► https://www.mdpi.com/authors/english.

MDPI Multidisciplinary Digital Publishing Institute

Basel, January 2020

Martyn Rittman, Ph.D.
English Editing Manager
englishediting@mdpi.com
https://www.mdpi.com/authors/english
