# Supplementary material for: Endothelium-Independent Vasodilatory Effects of Isodillapiolglycol Isolated from Ostericum citriodorum
Source: Molecules. 2020 Feb 17;25(4):885. doi: 10.3390/molecules25040885 (PMC7070945; doi:10.3390/molecules25040885)
Supplement: Supplementary file 1 [file molecules-25-00885-s001.zip › Supplementary Materials for compound details.docx]

The structure information of eight compounds which were obtained from the ethyl acetate extract of *O. citriodorum*

The ethyl acetate extract of *Ostericum citriodorum* was subjected to silica gel column using gradient mixtures of chloroform-methanol as eluents to obtain eight major fractions (Fr. A- Fr. H). Then, by Sephadex LH-20 columns or ODS column, Fr. B afford compound 1, Fr. C afford compound 2 and 3, Fr. E afford compound 4, Fr. F afford compound 5 and 6, Fr. G afford compound 7, Fr. H afford compound 8 (Table 1).

**Table 1.** The information of ethyl acetate extract of *O. citriodorum*

| **NO** | **Name** | **Structure** | [**Molecular**](javascript:;)  [**formula**](javascript:;) | **Molecule weight** |
| --- | --- | --- | --- | --- |
| 1* | 8-(3,7-Dimethyl-octa-2,6-dienyl)-7-hydroxy-6-mehtoxy-chromen-2-one |  | C_20_H_24_O_4_ | 329.17 |
| 2 | Decursidin |  | C_24_H_26_O_7_ | 426.46 |
| 3 | 9-angeloyloxy-10-senecioyloxy-9,10-dihydroxanthyletin |  | C_24_H_26_O_7_ | 426.46 |
| 4 | 6,7-Dimethoxy-1,3-benzodioxole-4-methanol |  | C_10_H_12_O_5_ | 212.20 |
| 5 | Isodillapiolglycol |  | C_12_H_16_O_6_ | 256.25 |
| 6 | Nodakenetin |  | C_14_H_14_O_4_ | 246.26 |
| 7 | Lariciresinol |  | C_20_H_24_O_6_ | 360.40 |
| 8* | Ostercitriodin A |  | C_20_ H_26_O | 362.16 |

*: New compounds.

*Compound 1 (8-(3,7-Dimethyl-octa-2,6-dienyl)-7-hydroxy-6-mehtoxy-chromen-2-one)*: Light yellow gum; C_20_H_24_O_4_; HRESIMS *m/z* 329.1745 ([M + H] ^+^ calcd for C_20_H_25_O_4_, 329.1747). ^1^H-NMR (CDCl_3_, 400 MHz) *δ*_H_ 7.57 (1H, d, *J* = 9.5 Hz, H-4), 6.25 (1H, d, *J* = 9.5 Hz, H-3), 6.25 (1H, s, H-5), 5.28 (1H, d, *J* = 6.8 Hz, H-10), 5.03 (1H, d, *J* = 6.8 Hz, H-15), 3.93 (3H, s, H-6-OCH_3_), 3.57 (2H, d, *J* = 7.3 Hz, H-9), 2.05 (2H, m, H-13), 1.96 (2H, m, H-14), 1.84 (3H, s, H-12), 1.61 (3H, s, H-17), 1.55 (3H, s, H-18). ^13^C-NMR (CDCl_3_, 100 MHz) *δ*_C_ 161.8 (C-2), 148.5 (C-7), 9) 147.6 (C-8a), 143.9 (C-4), 143.8 (C-6), 136.8 (C-11), 131.4 (C-16), 124.4 (C-15), 120.7 (C-10), 116.4 (C-8), 113.1 (C-4a), 111.3 (C-3), 105.2 (C-5), 56.4 (6-OCH3), 39.9 (C-13), 26.8 (C-14), 25.8 (C-17), 22. (C-, 17.8 (C-18), 16.4 (C-12). In the HMBC spectrum, the correlations between H-3 and C-2/ C-4a, between H-4 and C-2, C-4a, C-8a, between H-5 and C-2, C-4a, C-8a, C-6, C-7, between H-9 and C-7, C-8, C-8a, indicating that the structural fragment of coumarin can be determined. In addition, the HMBC correlations between H-9 and C-7, C-8, C-8a, between H-10 and C-8, suggested C-9 is connected to C-8. In addition, by ROESY, the correlations between H-5 and C-2/ 8-OCH_3_, between H-9 and H-12, between H-10 and H-13. Based on the above information of ^1^H-NMR, ^13^C-NMR, COSY, HSQC, HMBC and ROESY, we determined that this compound is 8-(3,7-Dimethyl-octa-2,6-dienyl)-7-hydroxy-6-mehtoxy-chromen-2-one.

**Figure 1.** Key ^1^H-^1^H COSY and HMBC correlations of compound 1.


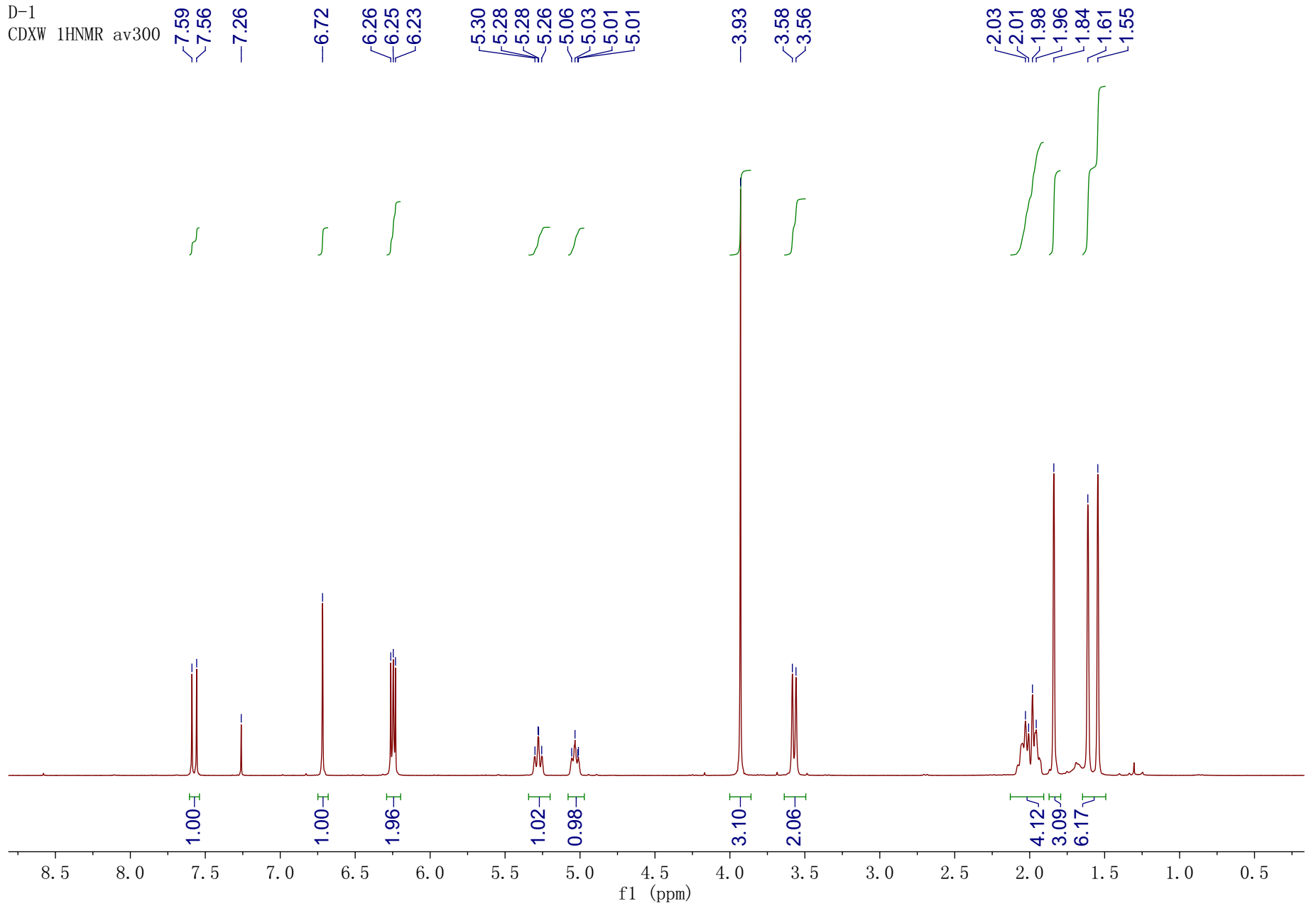
2

**Figure 2.** ^1^H-NMR Spectrum of Compound 1


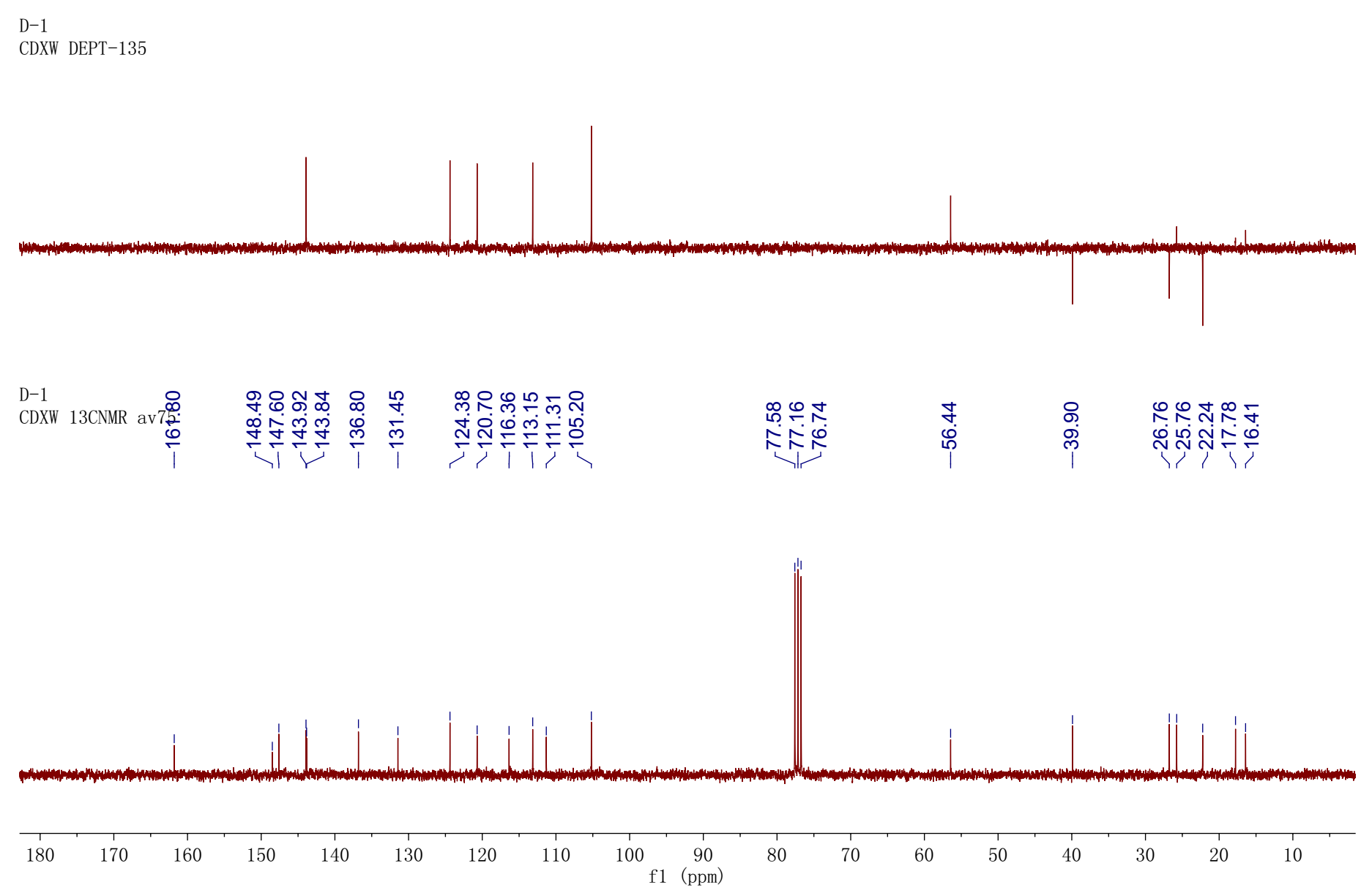


**Figure 3.** ^13^C-NMR Spectrum of Compound 1


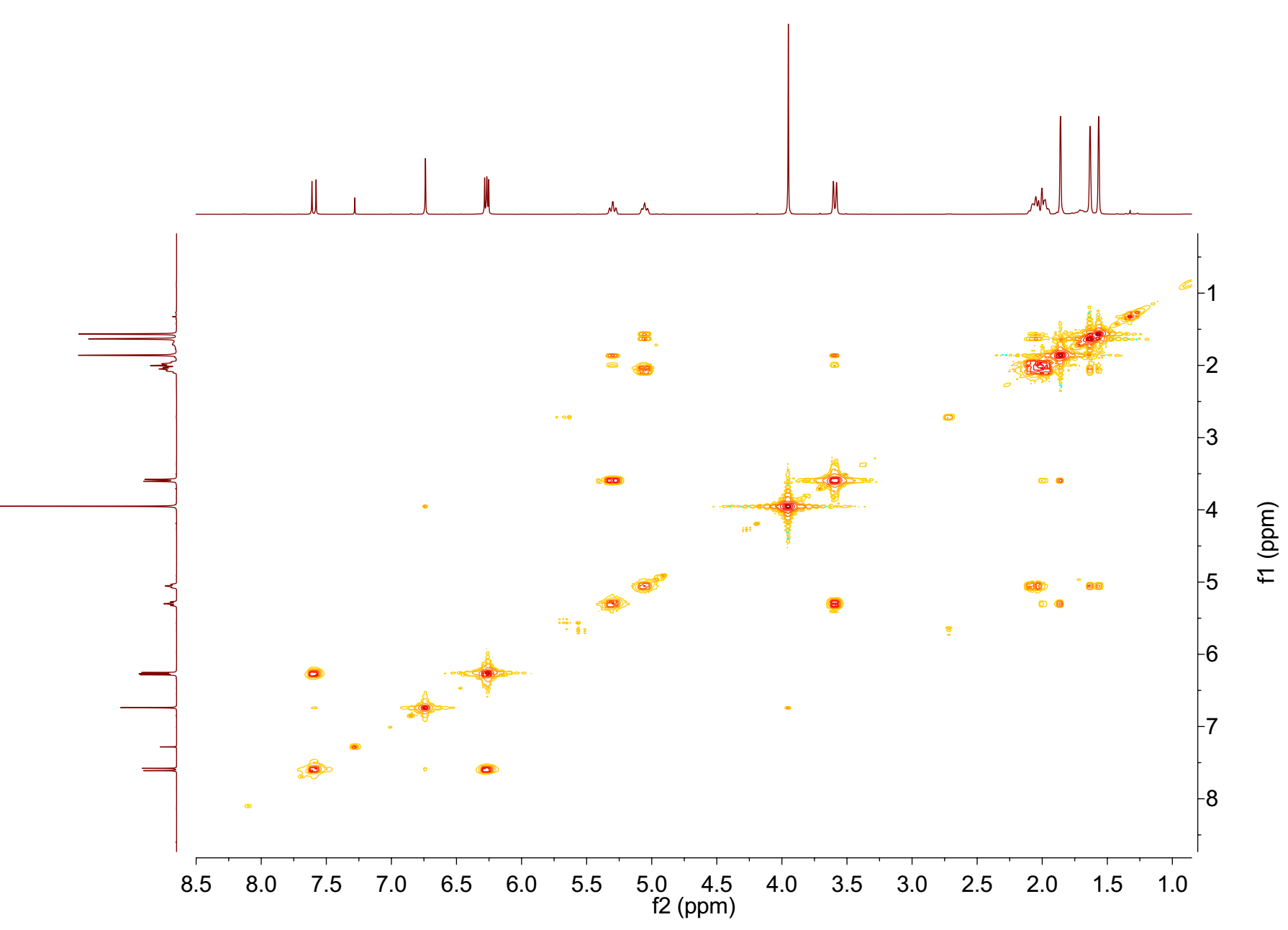


**Figure 4.** COSY Spectrum of Compound 1


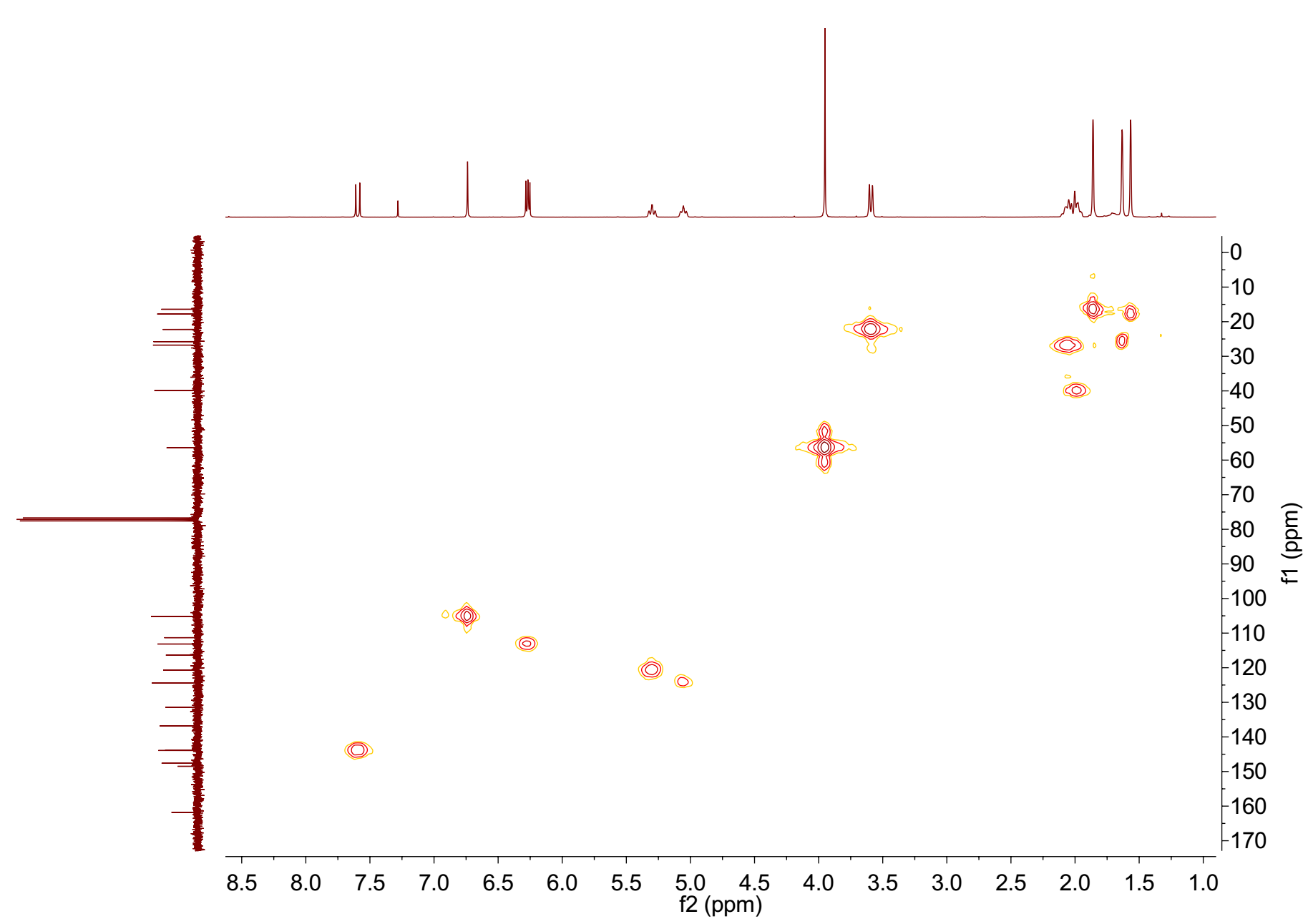


**Figure 5.** HSQC Spectrum of Compound 1


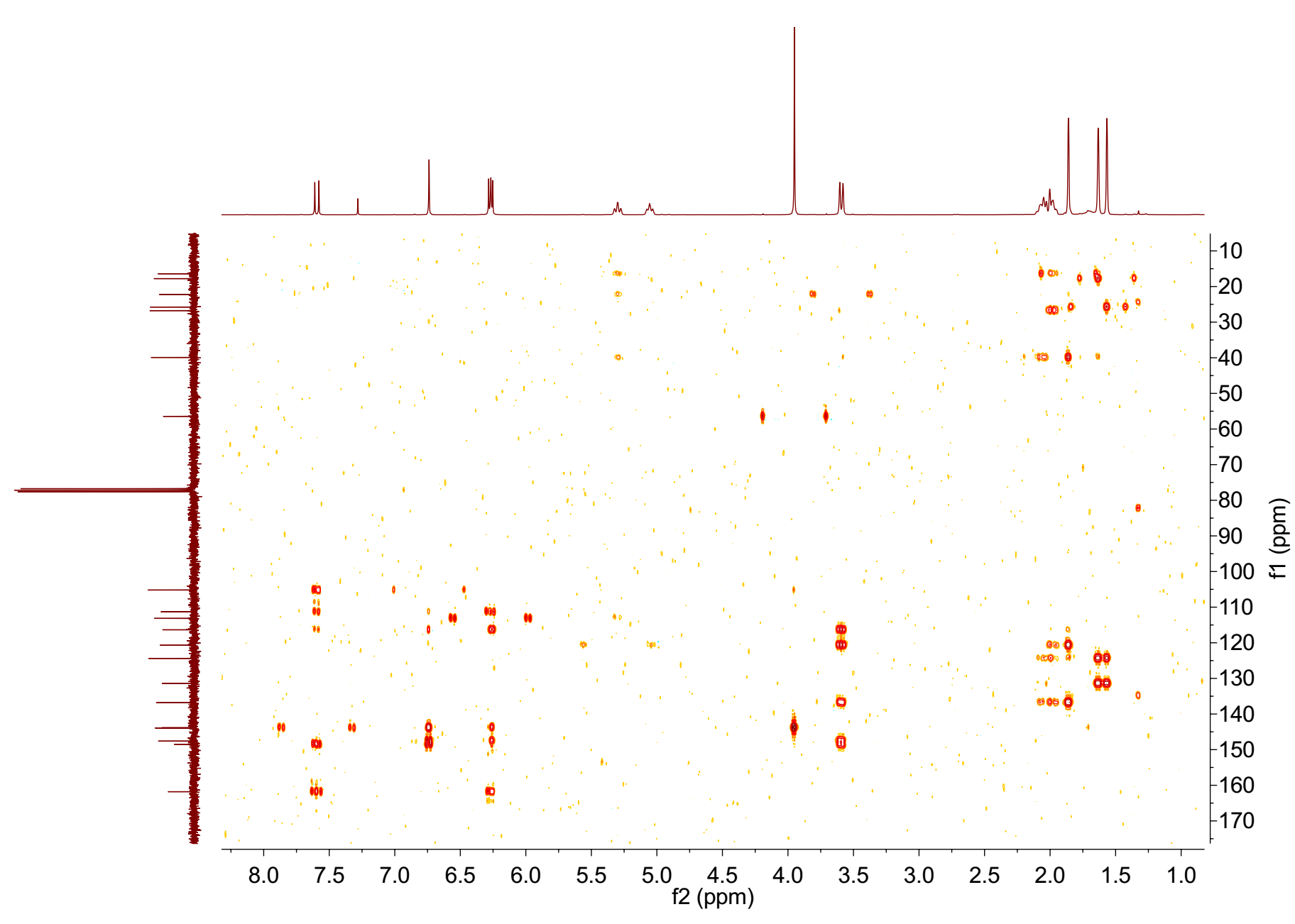


**Figure 6.** HMBC Spectrum of Compound 1


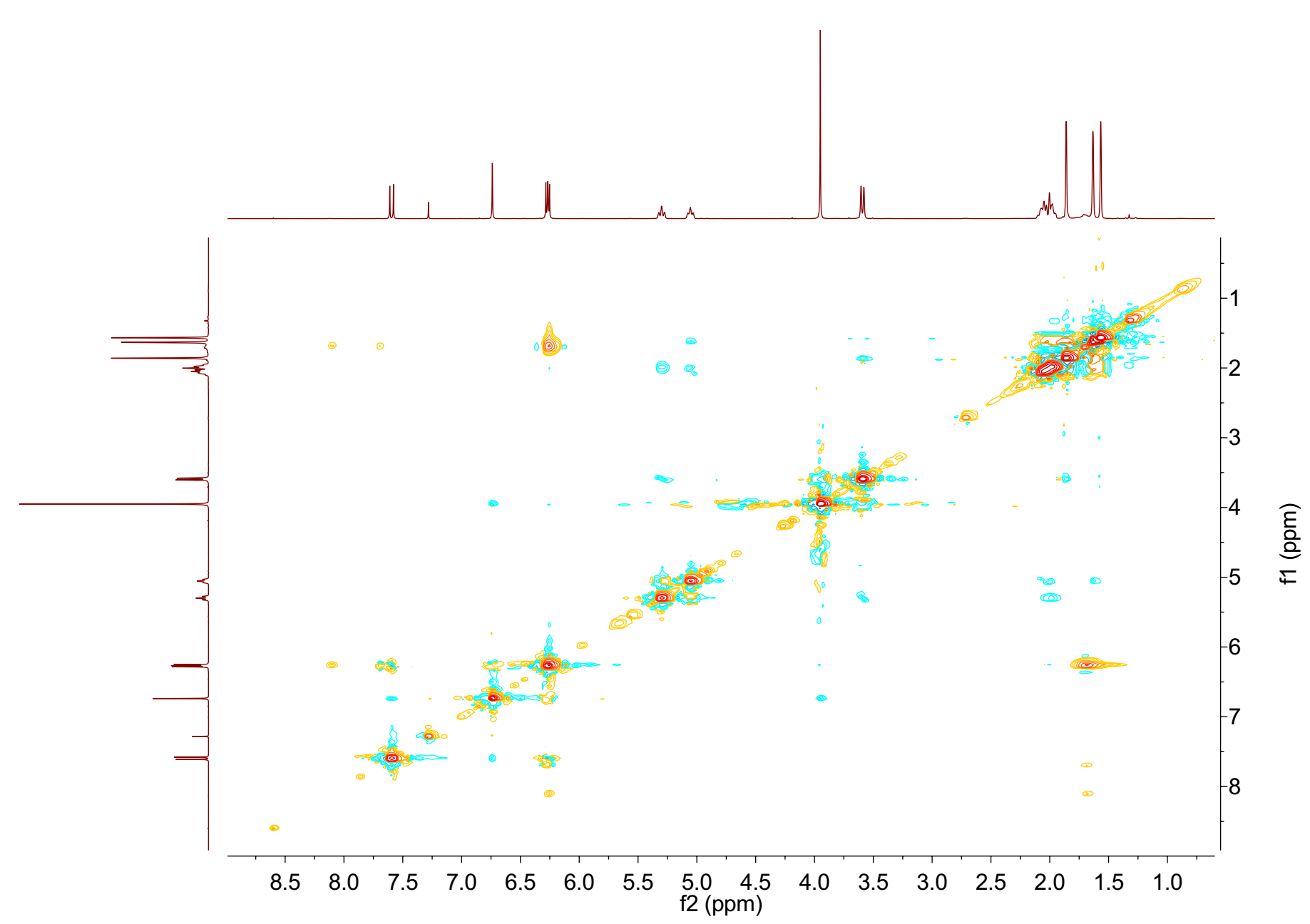


**Figure 7.** ROESY Spectrum of Compound 1

*Compound 2 (Decursidin)*: Light yellow gum, C_24_H_26_O_7_; HRESIMS *m/z*: 427.1750 [M + H] ^+^ (calcd for C_24_H_26_O_7_, 427.1751). ^1^H-NMR (CDCl_3_, 400 MHz): *δ*_H_ 7.58 (1H, d, *J* = 9.5 Hz, H-4), 6.23 (1H, d, *J* = 9.5 Hz, H-3), 7.37 (1H, s, H-5), 6.79 (1H, s, H-8), 5.68 (1H, overlap, H-2’), 5.68 (1H, overlap, H-2’’), 6.04 (1H, d, *J* = 5.7 Hz, H-10), 5.26 (1H, d, *J* = 5.7 Hz, H-9), 2.21 (3H, brd, *J* = 1.0 Hz, H-5’’), 2.15 (3H, brd, *J* = 1.0 Hz, H-5’), 1.92 (3H, brd, *J* = 1.0 Hz, H-4’’), 1.89 (3H, brd, *J* = 1.0 Hz, H-4’), 1.45 (3H, s, H-12), 1.38 (3H, s, H-13). ^13^C-NMR (CDCl_3_, 100 MHz) : δ_C_ 166.1 (C-1’), 165.1 (C-1’’), 161.0 (C-2), 159.5 (C-3’’), 159.4 (C-3’), 156.4 (C-7), 155.4 (C-8a), 143.4 (C-4), 129.5 (C-5), 117.3 (C-6), 115.3 (C-2’’), 115.1 (C-2’), 113.8 (C-3), 113.4 (C-4a), 105.0 (C-8), 78.1 (C-11), 71.3 (C-10), 66.3 (C-9), 27.7 (C-4’), 27.7 (C-4’’), 25.1 (C-12), 22.7 (C-13), 20.6 (C-5’), 20.6 (C-5’’). Compared with the reported data [1], compound 2 was identified as Decursidin.


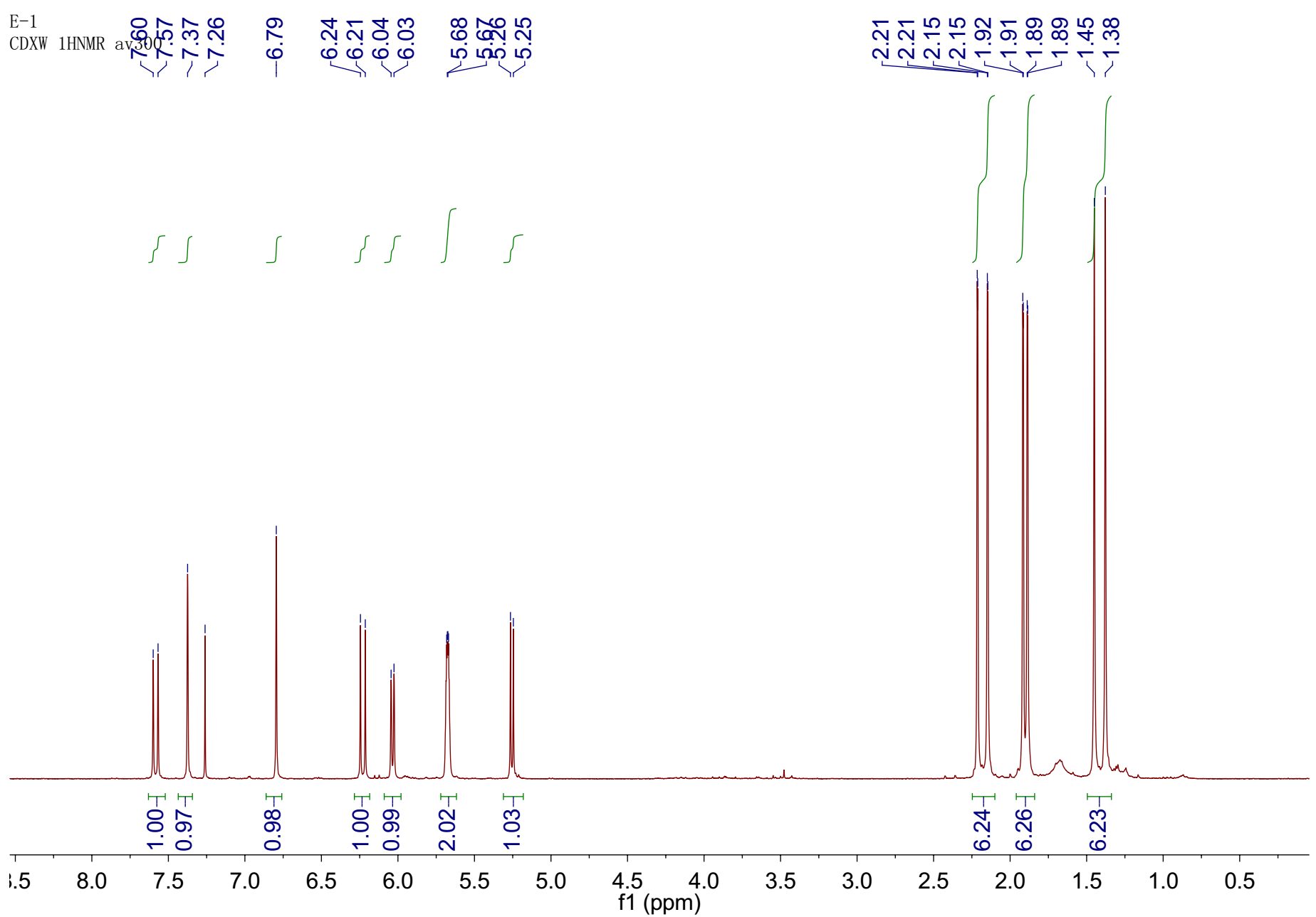


**Figure 8.** ^1^H-NMR Spectrum of Compound 2


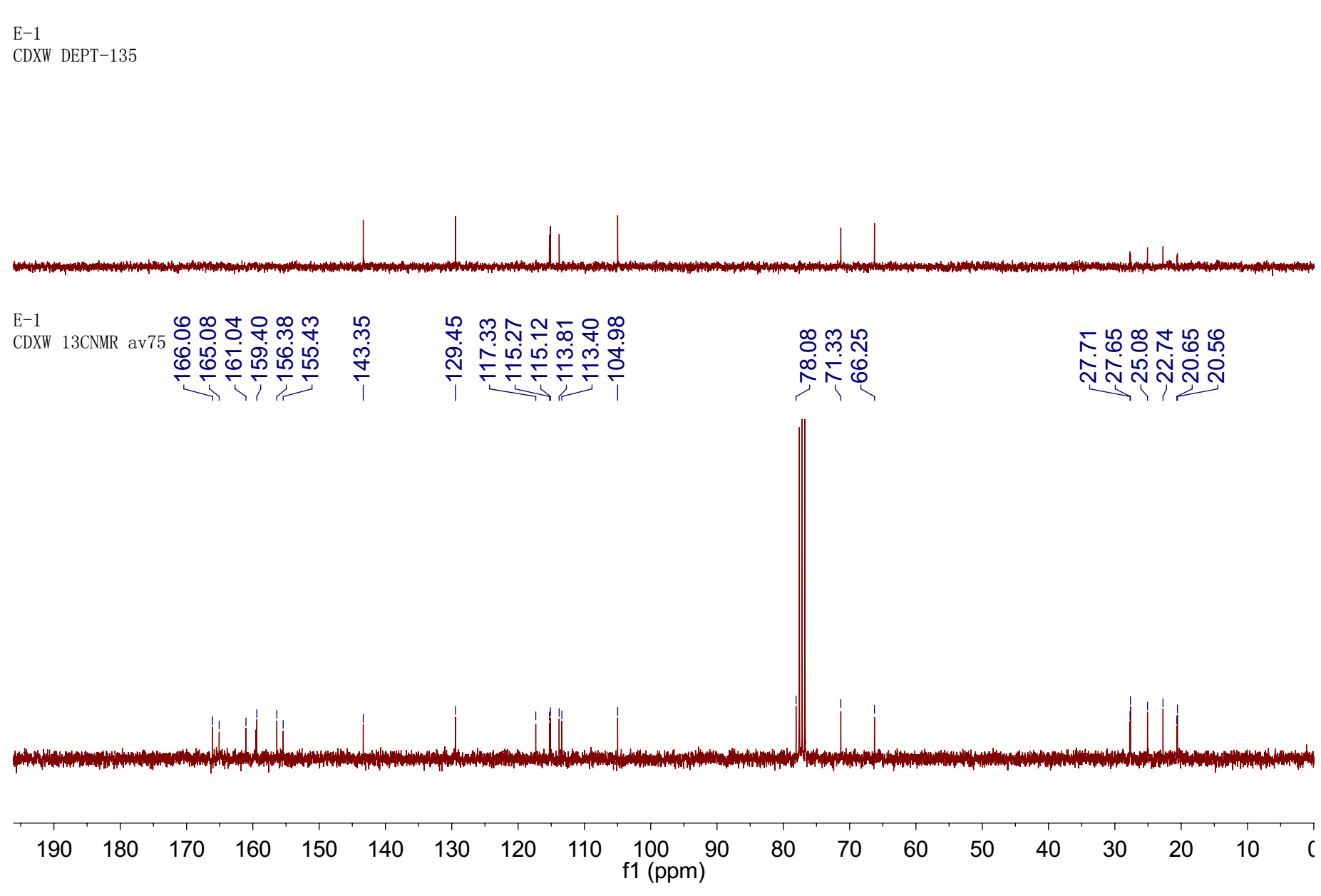


**Figure 9.** ^13^C-NMR Spectrum of Compound 2

*Compound 3 (9-angeloyloxy-10-senecioyloxy-9,10-dihydroxanthyletin)*: Light yellow gum, C_24_H_26_O*7*; HRESIMS *m/z* 427.1751 [M + H] ^+^ (calcd for C_24_H_27_O_7_, 427.1751).; ^1^H-NMR (CDCl3, 400 MHz) δH 7.58 (1H, d, J = 9.5 Hz, H-4), 6.23 (1H, d, J = 9.5 Hz, H-3), 7.35 (1H, s, H-5), 6.79 (1H, s, H-8), 6.11 (1H, m, H-3’’), 5.69 (1H, m, H-2’), 6.09 (1H, d, J = 6.3 Hz, H-10), 5.33 (1H, d, *J* = 6.3 Hz, H-9), 2.21 (3H, brd, *J* = 1.0 Hz, H-5’’), 1.90 (6H, overlap, H-4’,H-5’), 1.84 (3H, m, H-4’’), 1.46 (3H, s, H-12), 1.39 (3H, s, H-13). Compared with the reported data [2], compound 3 was identified as 9-angeloyloxy-10-senecioyloxy-9,10-dihydroxanthyletin. And we first reported it ^13^C-NMR (CDCl_3_, 100 MHz) date: *δ*_C_ 166.5 (C-1’), 166.1 (C-1’’), 161.0 (C-2), 159.7 (C-3’), 156.3 (C-7), 155.4 (C-8a), 143.3 (C-4), 139.7 (C-3’’), 129.1 (C-5), 127.1 (C-2’’), 117.4 (C-6), 115.2 (C-2’), 113.9 (C-3), 113.4 (C-4a), 104.9 (C-8), 78.2 (C-11), 72.1 (C-10), 66.3 (C-9), 27.7 (C-4’), 25.3 (C-12), 22.5 (C-13), 20.7 (C-5’), 20.6 (C-5’’), 15.9 (C-4’’).


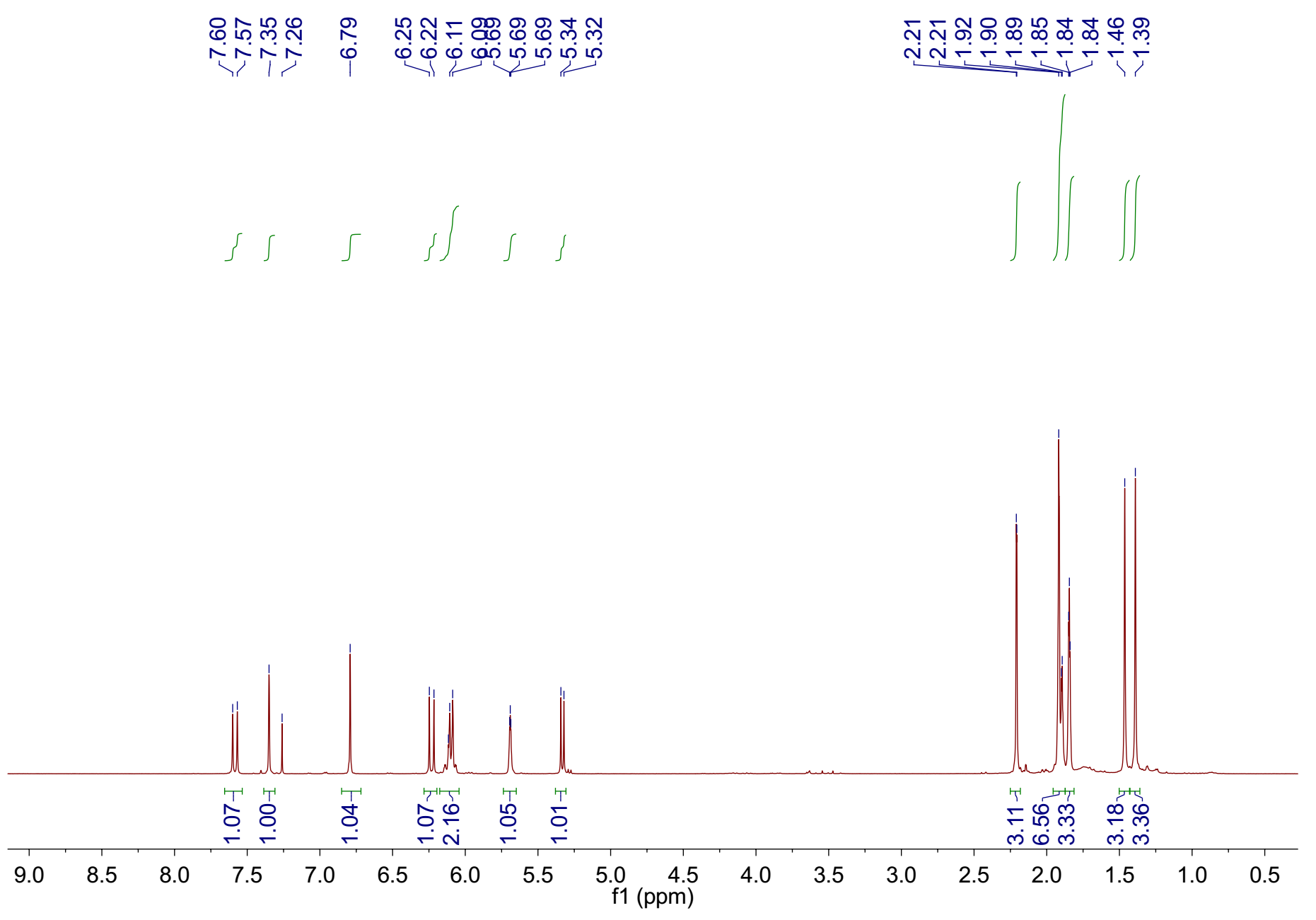


**Figure 10.** ^1^H-NMR Spectrum of Compound 3


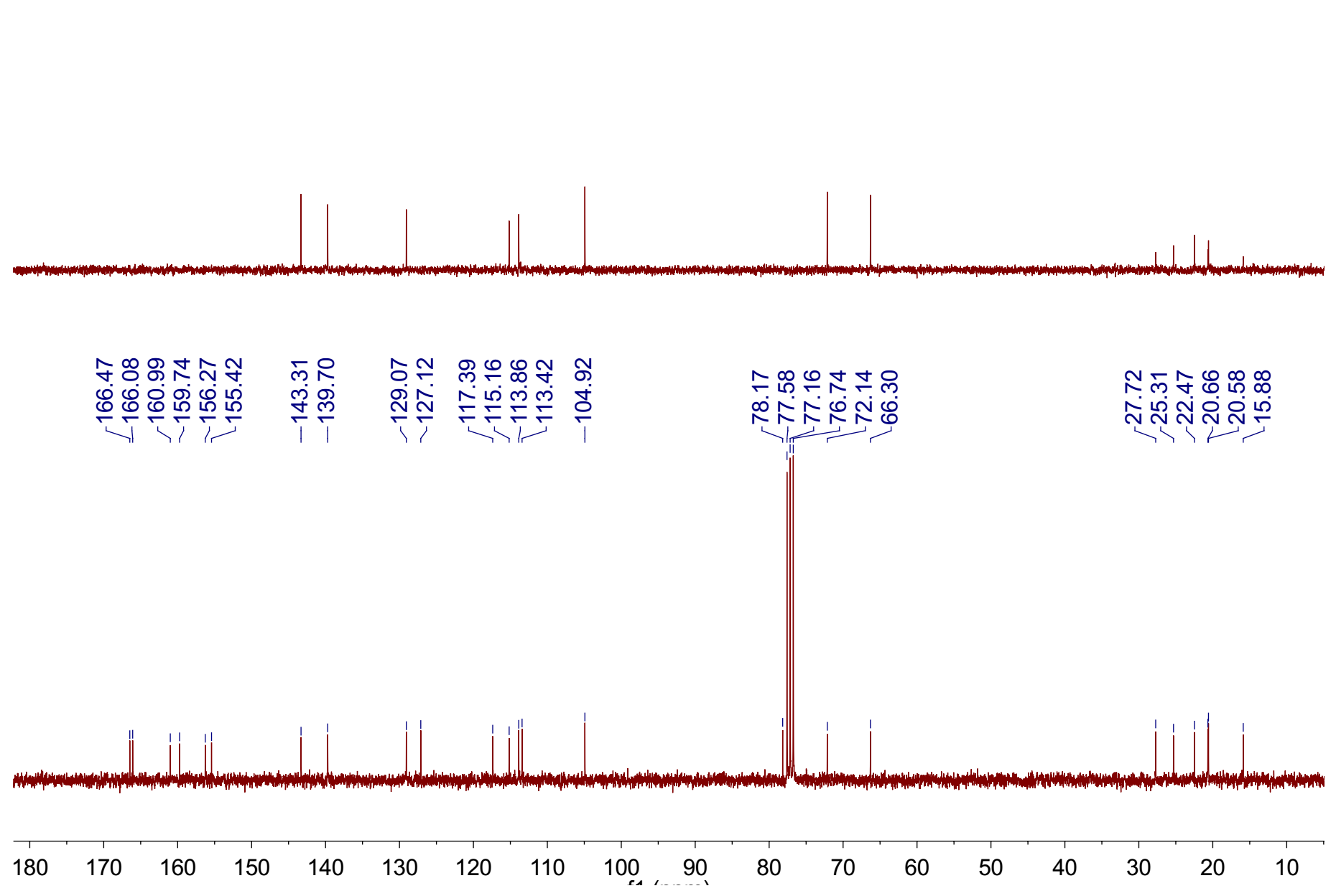


**Figure 11.** ^13^C-NMR Spectrum of Compound 3

*Compound 4 (6,7-Dimethoxy-1,3-benzodioxole-4-methanol)*: White amorphous powder, C_10_H_12_O_5_; ESI-MS (positive) *m/z*: 235 [M + Na] ^+^. ^1^H-NMR (CDCl_3_, 400 MHz) *δ*_H_ 6.48 (1H, s, H-6), 5.96 (2H, s, H-8), 4.57 (2H, s, H-7), 3.95 (3H, s, 4-OCH_3_), 3.85 (3H, s, 5-OCH_3_). Compared with the reported data [3], compound 4 was identified as 6,7-Dimethoxy-1,3-benzodioxole-4-methanol. And we first reported it ^13^C-NMR (CDCl3, 100 MHz) date δ_C_ 138.9 (C-5), 138.2 (C-3), 136.7 (C-2), 136.4 ( C-4), 126.1 (C-1), 107.8 (d, C-6), 101.8 ( C-8), 61.8 (C-7), 60.2 (4-OCH_3_), 57.0 (5-OCH_3_).


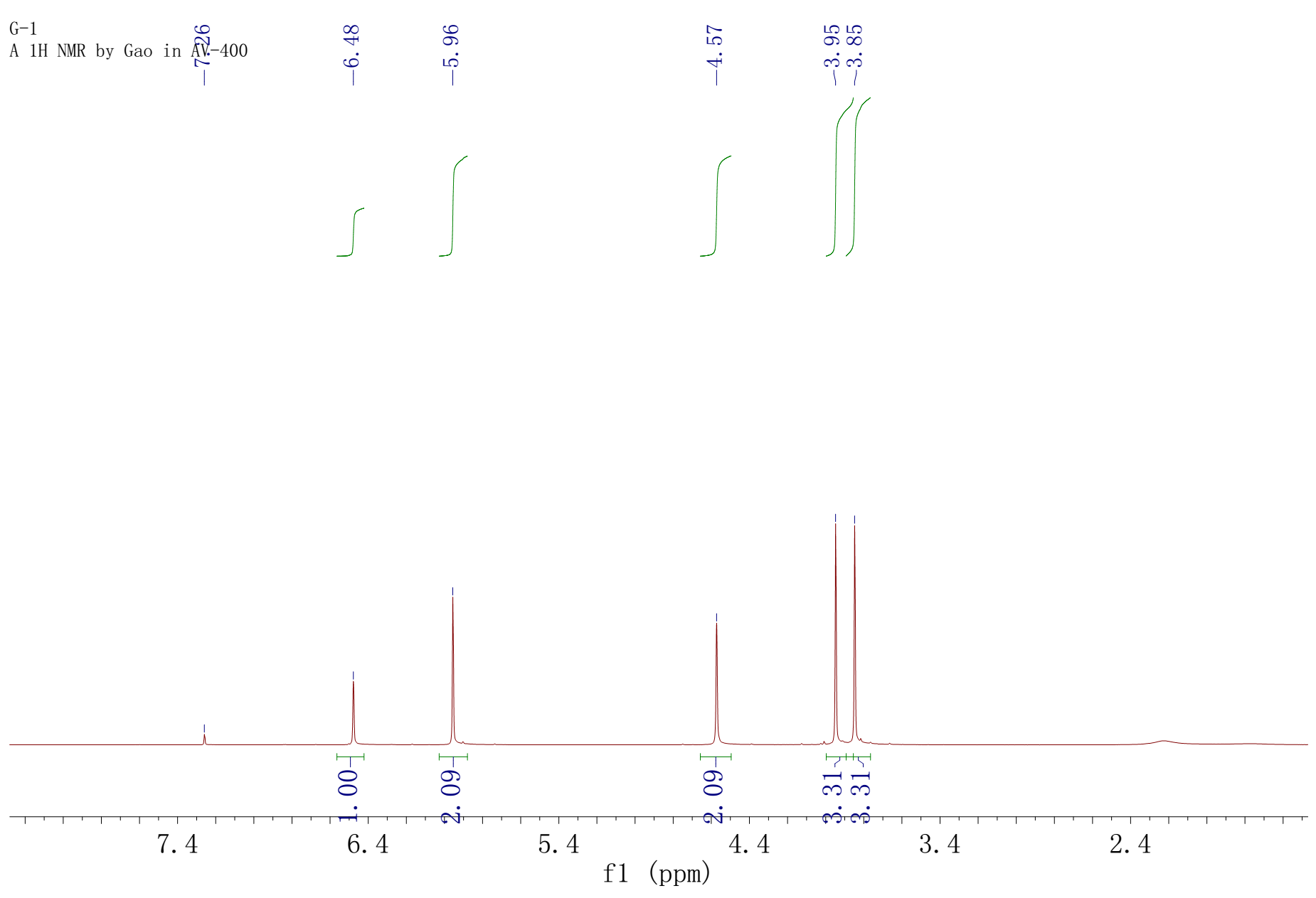


**Figure 12.** ^1^H-NMR Spectrum of Compound 4


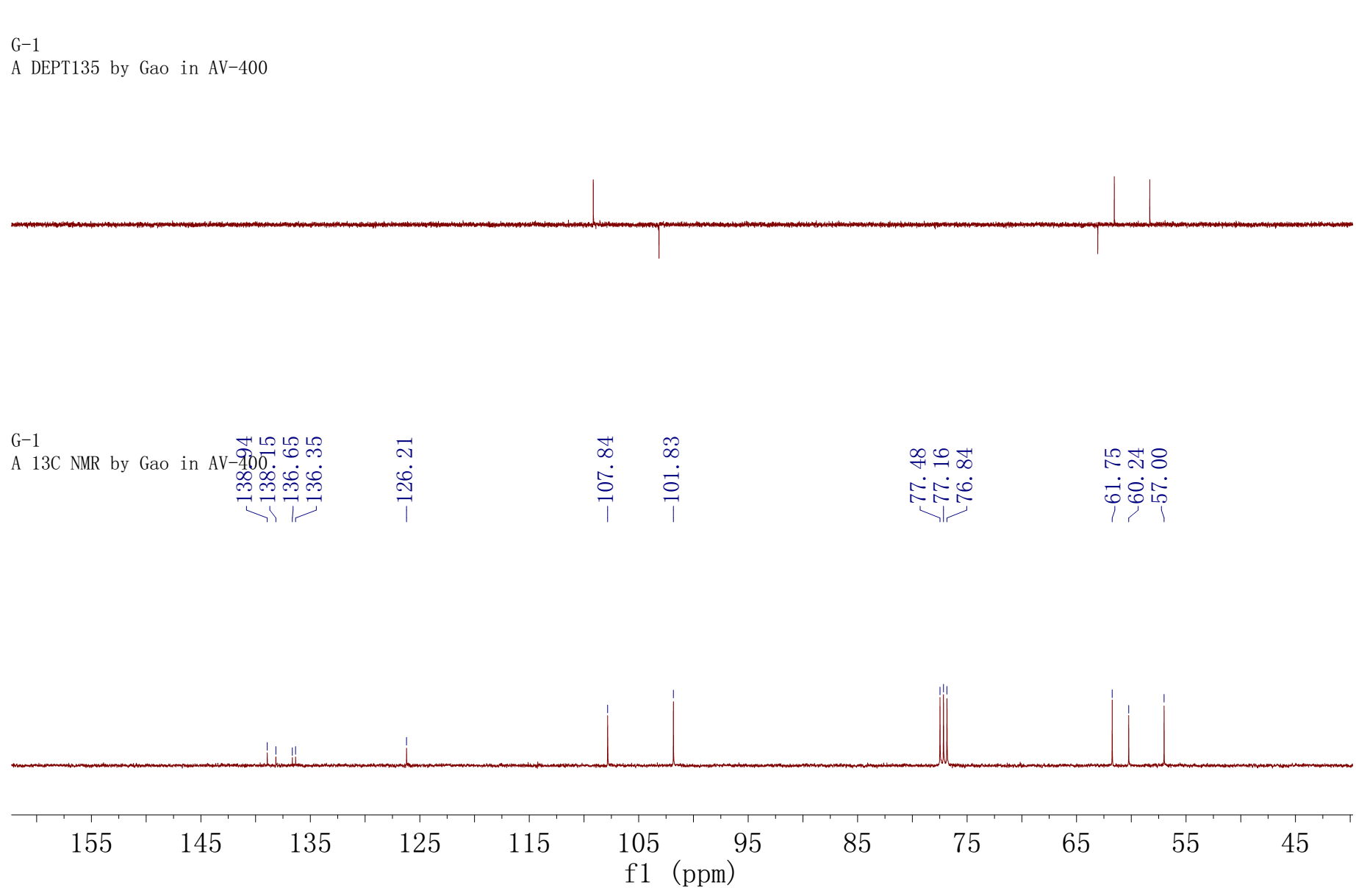


**Figure 13.** ^13^C-NMR Spectrum of Compound 4

*Compound 5* (isodillapiolglycol). The structural identification has been described in the manuscript and its related atlas information is shown here.


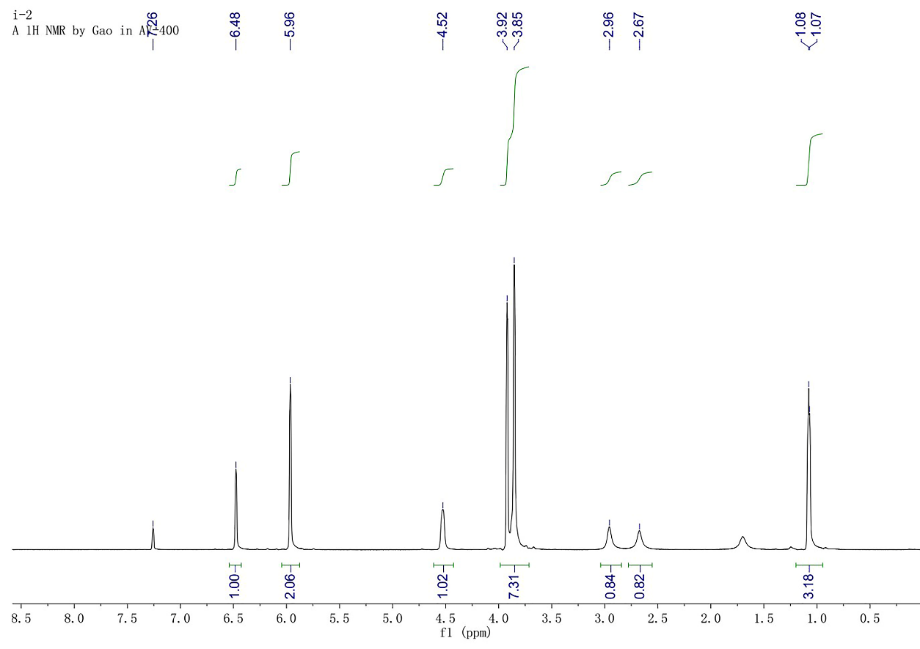


**Figure 14.** ^1^H-NMR Spectrum of Compound 5


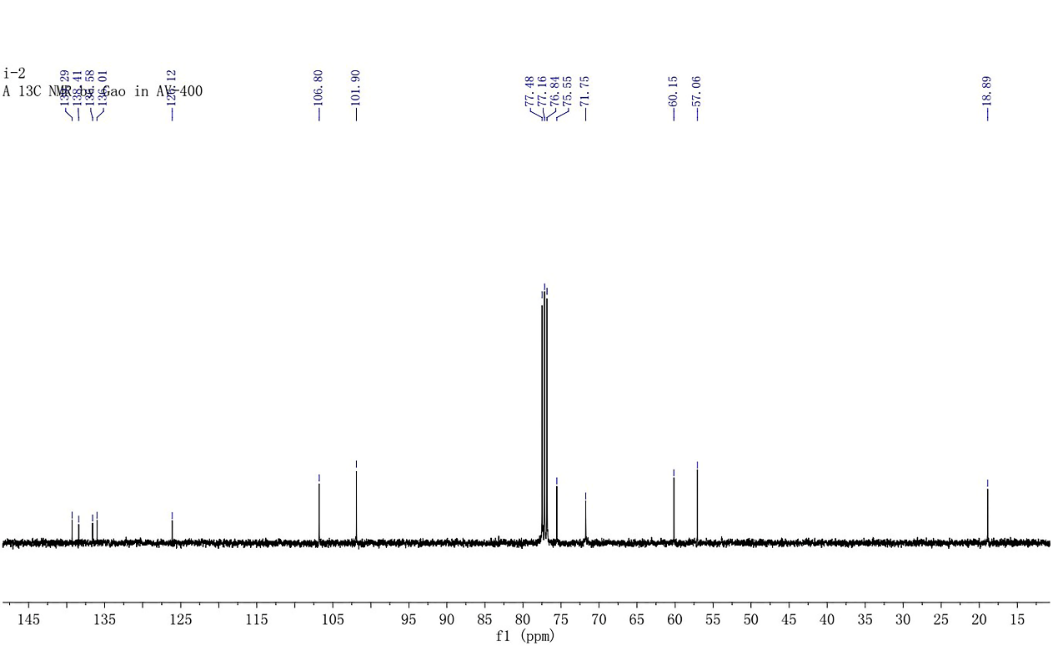


**Figure 15.** ^13^C-NMR Spectrum of Compound 5

**Figure 16.** ^1^H-^1^H COSY Spectrum of Compound 5

**Figure 17.** HSQC Spectrum of Compound 5

**Figure 18.** HMBC Spectrum of Compound 5

*Compound 6 (Nodakenetin)*: Light yellow gum, C_14_H_14_O_4_; HRESIMS *m/z* 247.0959 ([M + H] ^+^ calcd for C_14_H_15_O_4_, 247.0965). ^1^H-NMR (CDCl_3_, 400 MHz) *δ*_H_ 7.55 (1H, d, *J* = 9.5 Hz, H-4), 6.15 (1H, d, *J* = 9.5 Hz, H-3), 7.18 (1H, s, H-5), 6.66 (1H, s, H-8), 4.71 (1H, t, *J* = 8.8 Hz, H-10), 3.19 (2H, m, H-9), 1.35 (3H, s, H-12), 1.21 (3H, s, H-13). ^13^C-NMR (CDCl_3_, 100 MHz) *δ*_C_ 163.3 (C-7), 161.6 (C-2), 155.6 (C-8a), 143.9 (C-4), 125.3 (C-6), 123.5 (C-5), 112.8 (C-4a), 112.1 (C-3), 97.9 (C-3), 91.3 (C-10), 29.5 (C-9), 71.7 (C-11), 26.1 (C-12), 24.4 (C-13). Compared with the reported data [4], compound 6 was identified as Nodakenetin.

*
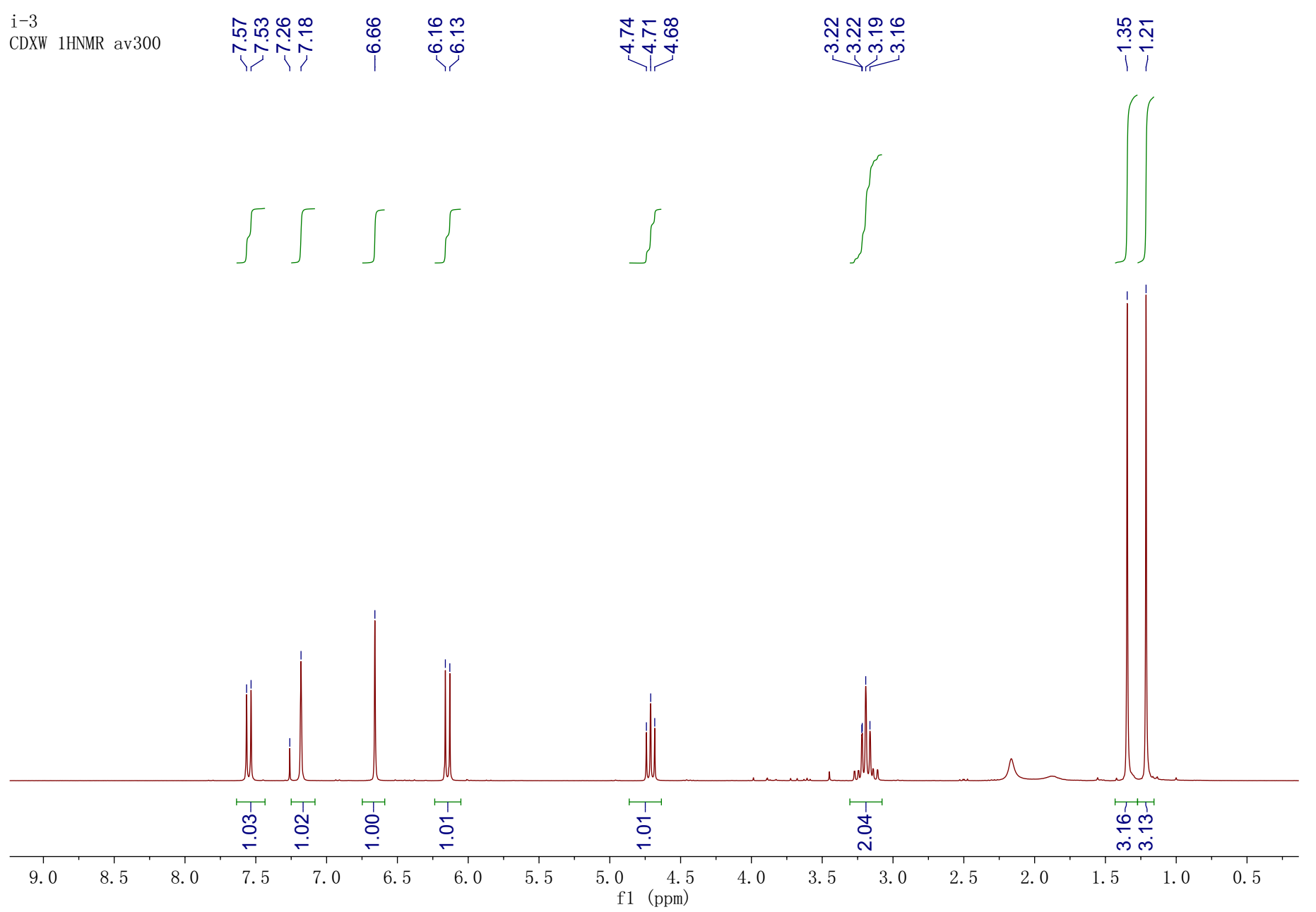
*

**Figure 18.** ^1^H-NMR Spectrum of Compound 6

*
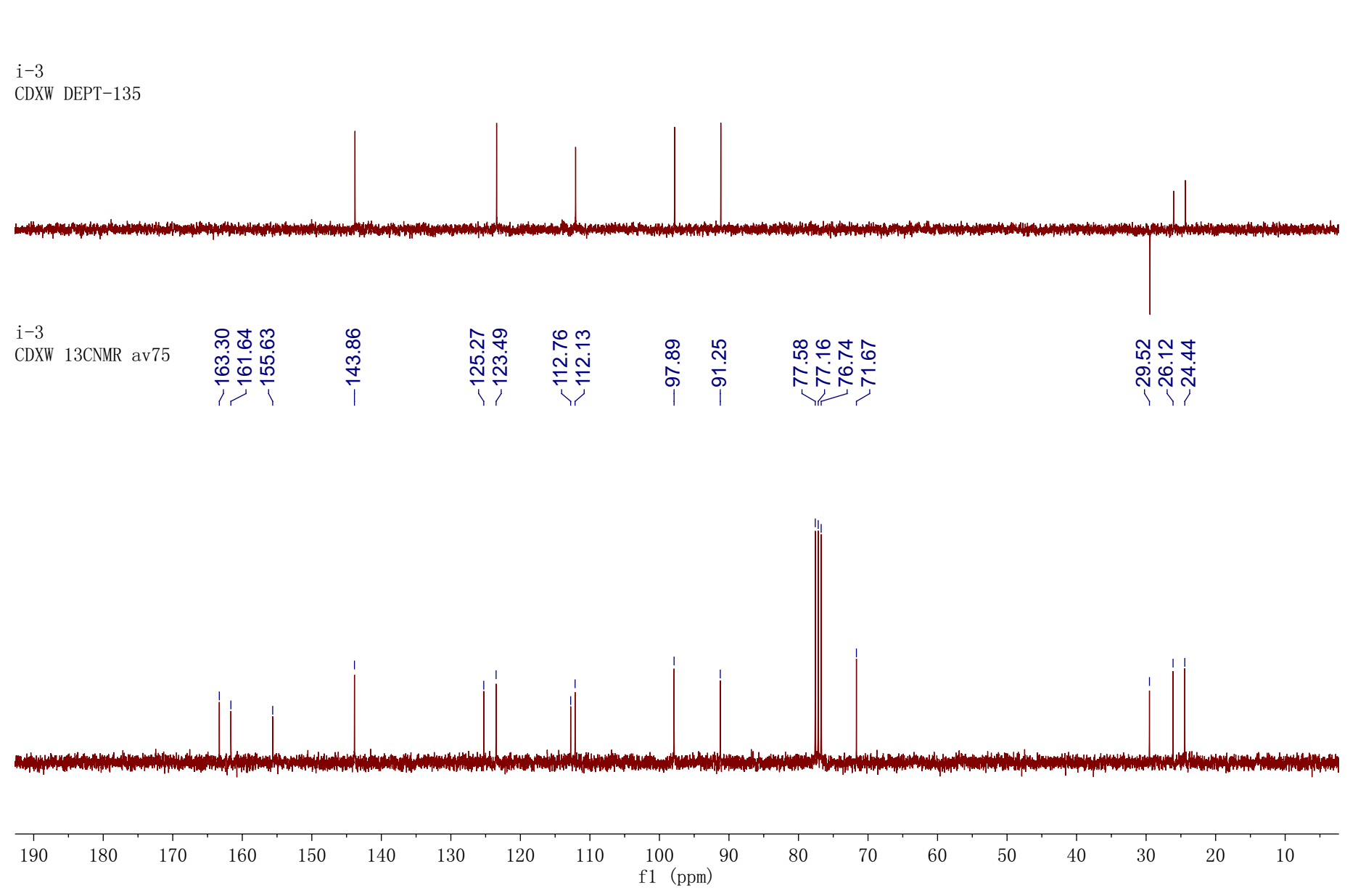
*

**Figure 19.** ^13^C-NMR Spectrum of Compound 6

*Compound 7 ((+)-Lariciresinol)*: Light yellow gum; C_20_H_24_O_6_; ESI-MS (positive) *m/z*: 235 [M + Na] ^+^. ^1^H-NMR (CD3OD, 400 MHz) *δ*_H_ 6.91 (1H, brs), 6.77 (1H, overlap), 6.79 (1H, d, *J* = 1.5 Hz), 6.72 (1H, d, *J* = 8.0 Hz), 6.64 (1H, dd, *J* = 8.0, 1.5 Hz), 4.74 (1H, d, *J* = 6.9 Hz), 3.98 (1H, dd, *J* = 8.2, 6.6 Hz), 3.83 (1H, overlap), 3.72 (1H, dd, *J* = 8.3, 5.9 Hz), 3.63 (1H, dd, *J* = 11.0, 6.4 Hz), 3.83 (3H, s), 3.82 (3H, s), 2.92 (1H, dd, *J* = 13.4, 4.7 Hz), 2.72 (1H, m), 2.48 (1H, dd, *J* = 13.1, 11.5 Hz), 2.37 (1H, m). ^13^C-NMR (CD3OD, 100 MHz) *δ*_C_ 149.0 (C-3/4'), 147.0 (C-4), 145.8 (C-3'), 135.7 (C-1), 133.5 (C-1'), 122.2 (C-6'), 119.8 (C-6), 116.2 (C-5'), 116.0 (C-5), 113.4 (C-2'), 110.6 (C-2), 83.0 (C-7'), 73.5 (C-9'), 60.4 (C-9'), 56.3 (C-10/10'), 54.0 (C-8'), 43.8 (C-8), 33.6 (C-7). Compared with the reported data [5], compound 7 was identified as (+)-Lariciresinol*.*


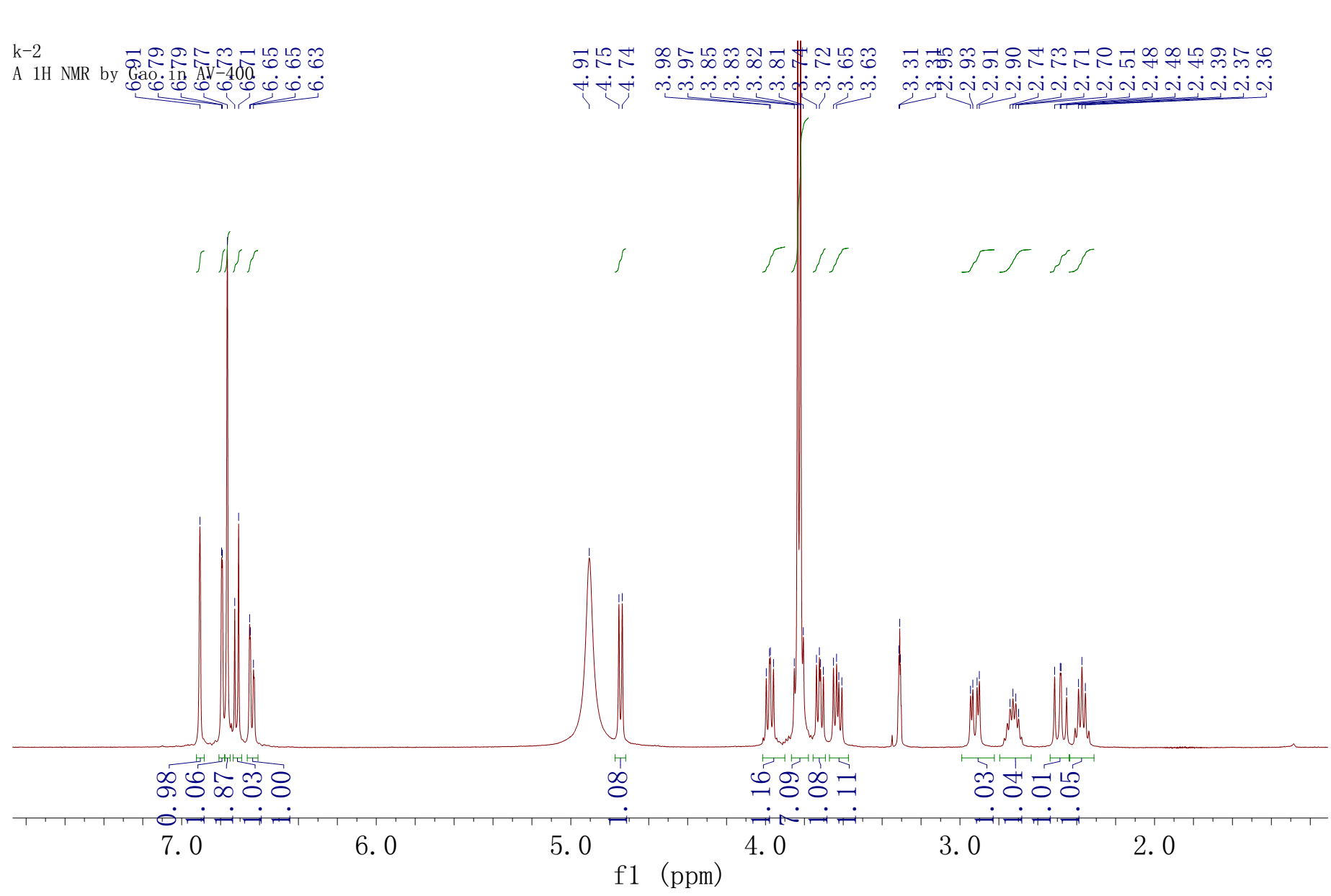
 **Figure 20.** ^1^H-NMR Spectrum of Compound 7


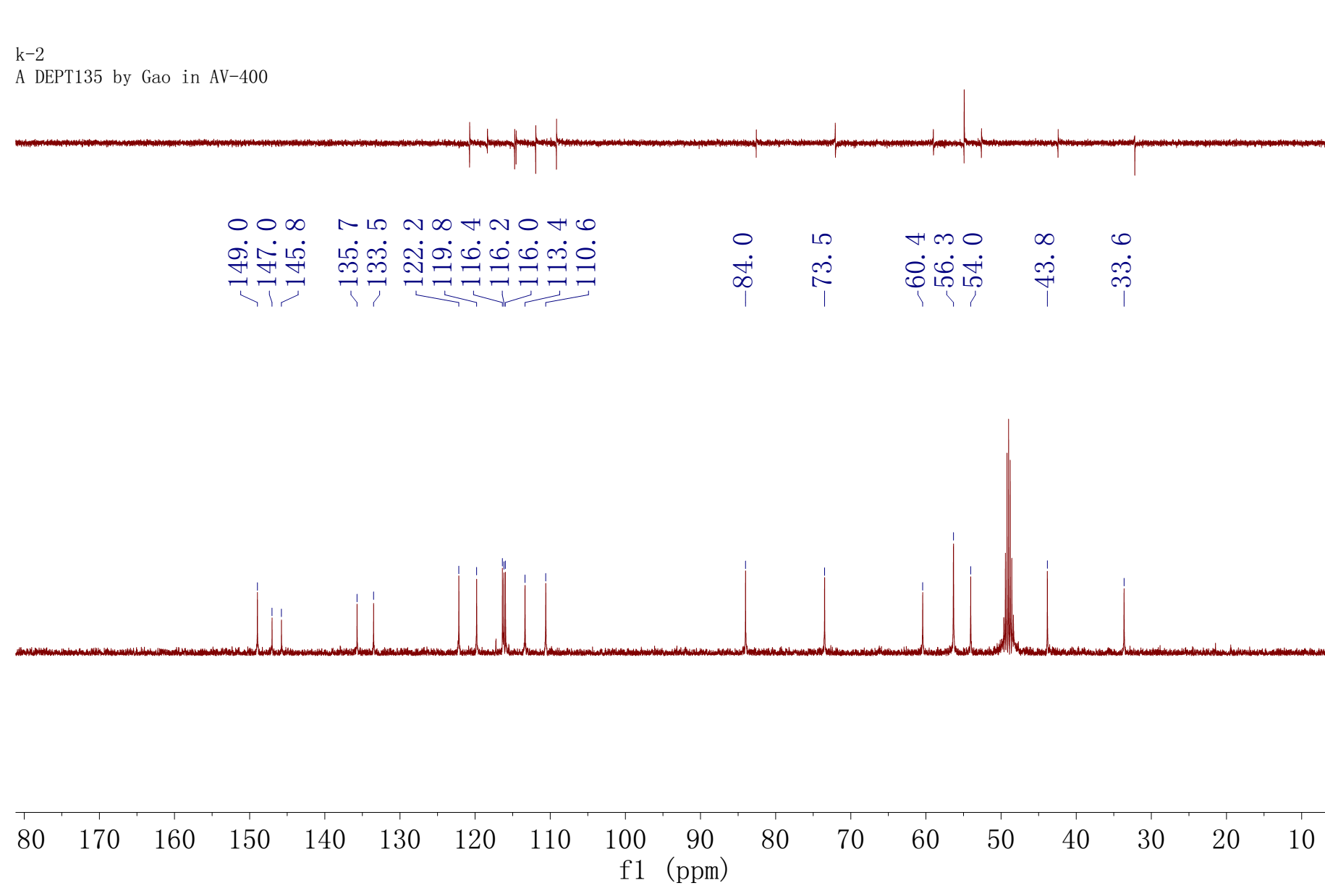


**Figure 21.** ^13^C-NMR Spectrum of Compound 7

*Compound 8 (Ostercitriodin A)*: Light yellow gum; C_20_H_26_O_6_, HRESIMS *m/z* 385.1628([M + Na] ^+^ calcd for C_20_H_26_O_6_, 385.1622). ^1^H-NMR (CD3OD, 400 MHz) *δ*_H_ 7.80 (1H, d, J = 9.4 Hz, H-4), 6.17 (1H, d, J = 9.4 Hz, H-3), 6.93 (1H, s, H-5), 5.31 (1H, d, J = 7.0 Hz, H-10), 3.90 (3H, s, H-8-OCH_3_), 3.52 (2H, d, J = 7.1 Hz, H-9), 3.19 (1H, dd, J = 10.4, 1.1 Hz, H-15), 2.23 (1H, m, Ha-13), 2.01 (1H, m, Hb-13), 1.70 (1H, m, Ha-14), 1.32 (1H, m, Hb-14), 1.85 (3H, s, H-12), 1.11 (3H, s, H-17), 1.08 (3H, s, H-18). ^13^C-NMR (CD3OD, 100 MHz) *δ*_C_ 164.3 (C-2), 150.5 (C-7), 149.5 (C-8a), 146.6 (C-4), 146.6 (C-6), 137.0 (C-11), 122.7 (C-10), 117.2 (C-8), 112.2 (C-4a), 112.1 (C-3), 107.0 (C-5), 78.9 (C-15), 73.8 (C-16), 56.7 (8-OCH3), 37.8 (C-13), 30.6 (C-14), 25.7 (C-17), 24.9 (C-18), 23.0 (C-9), 16.6 (C-12). In the HMBC spectrum, the correlations between H-3 and C-2/ C-4a, between H-4 and C-2, C-4a, C-8a, between H-5 and C-4, C-4a, C-8a, C-6, C-7, between H-9 and C-7, C-8, C-8a, indicating that the structural fragment of coumarin can be determined. In addition, the HMBC correlations between H-9 and C-7, C-8, C-8a, between H-10 and C-8, suggested C-9 is connected to C-8. Furthermore, the HMBC correlations between H-12 and C-10, C-11, C-13, between H-9 and H-14, H-11, between H-17/H-18 and C-15, C-16. Based on the above information of ^1^H-NMR , ^13^C-NMR , HMBC and HSQC, we determined that this compound was Ostercitriodin A.

**Figure 22.** Key ^1^H-^1^H COSY and HMBC correlations of compound 8.


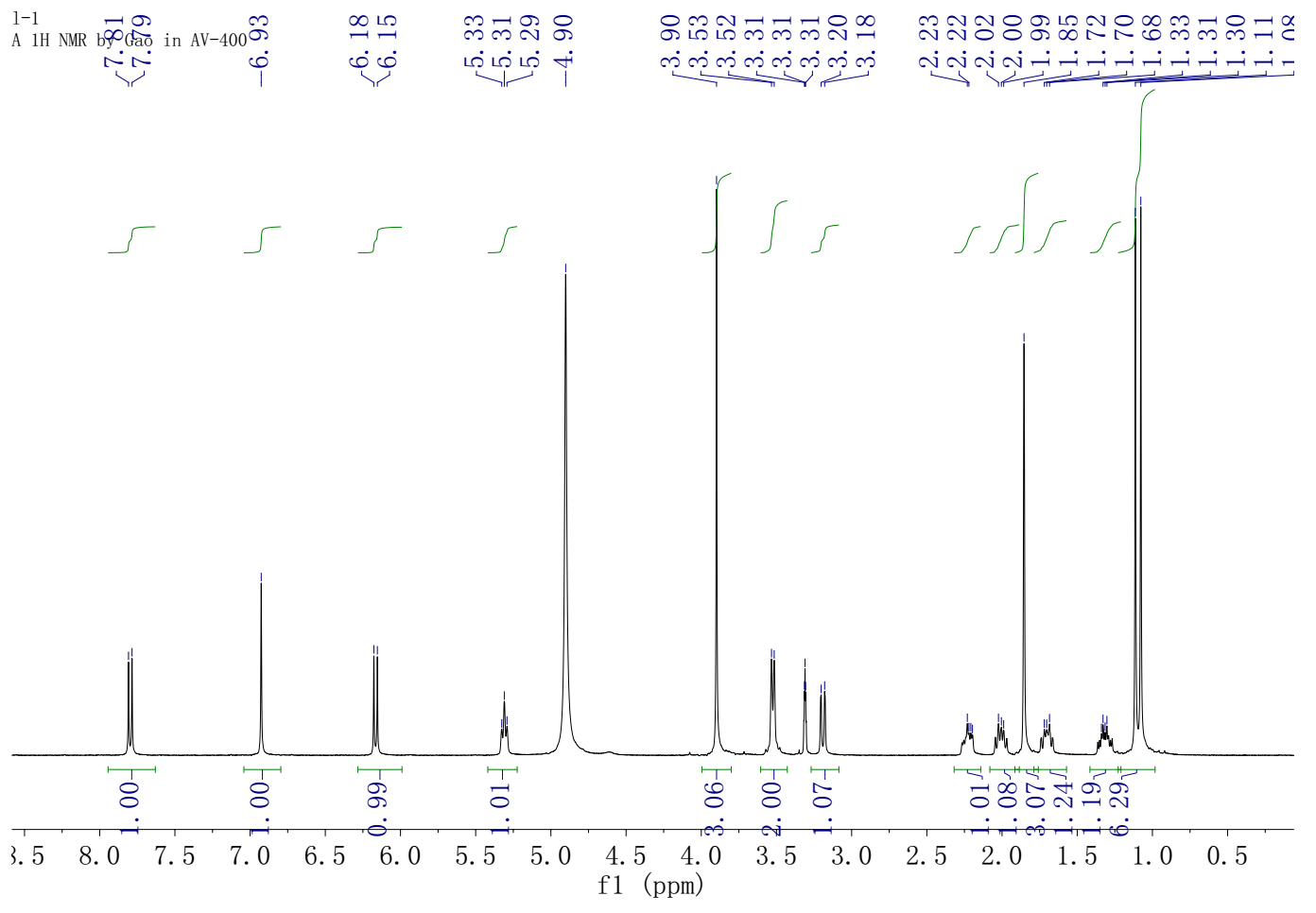


**Figure 23.** ^1^H-NMR Spectrum of Compound 8


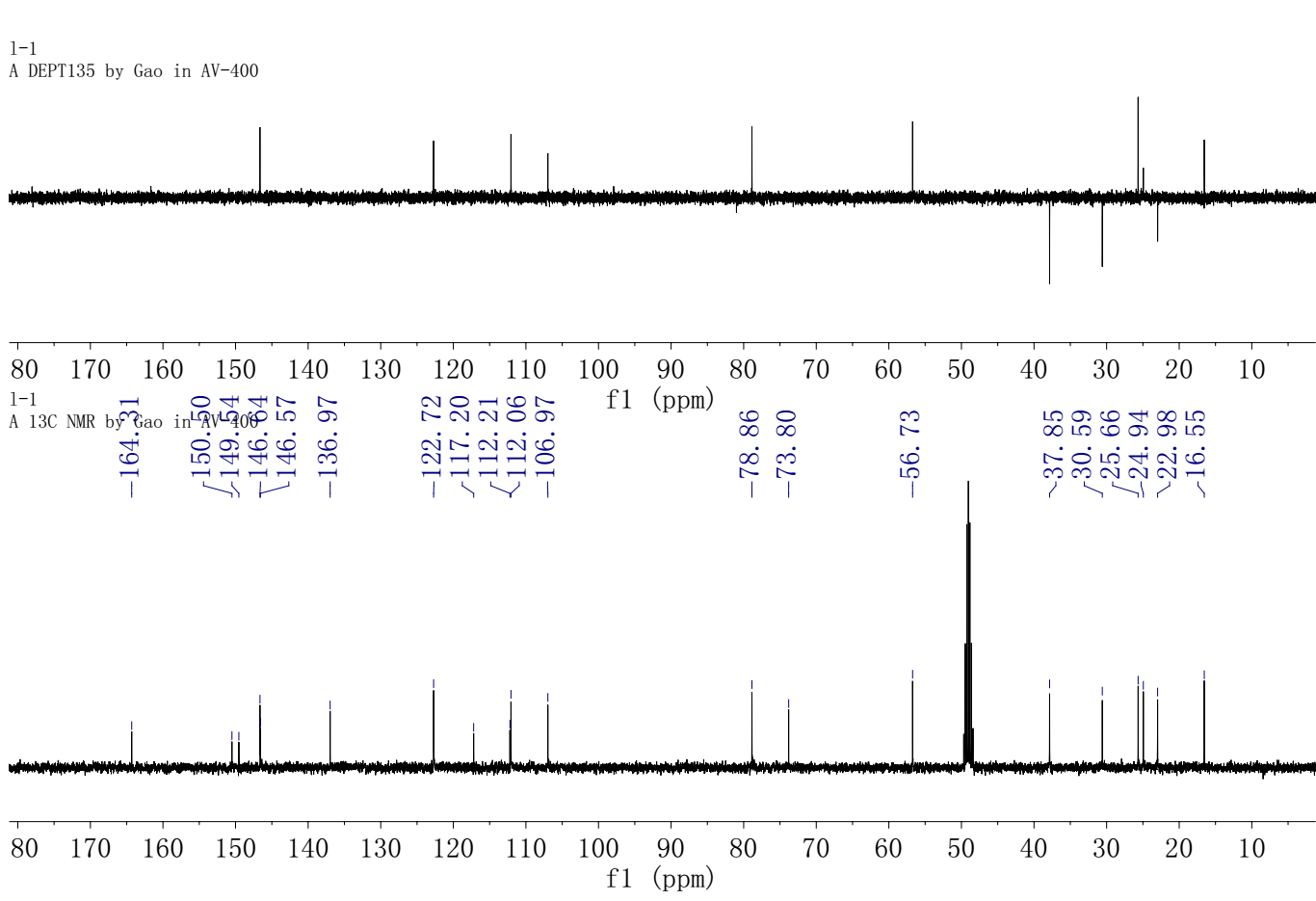


**Figure 24.** ^13^C-NMR Spectrum of Compound 8


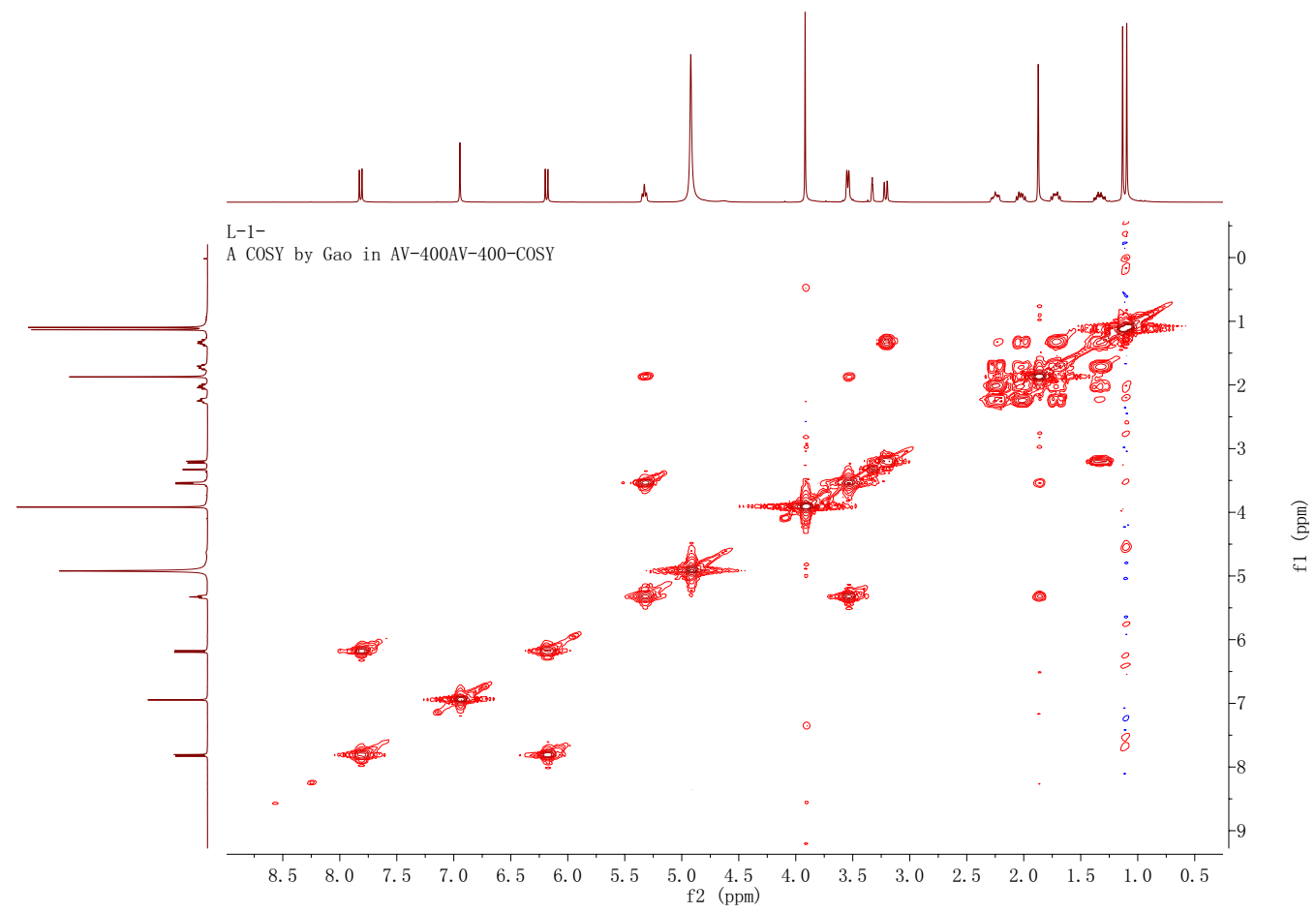


**Figure 25.** COSY Spectrum of Compound 8


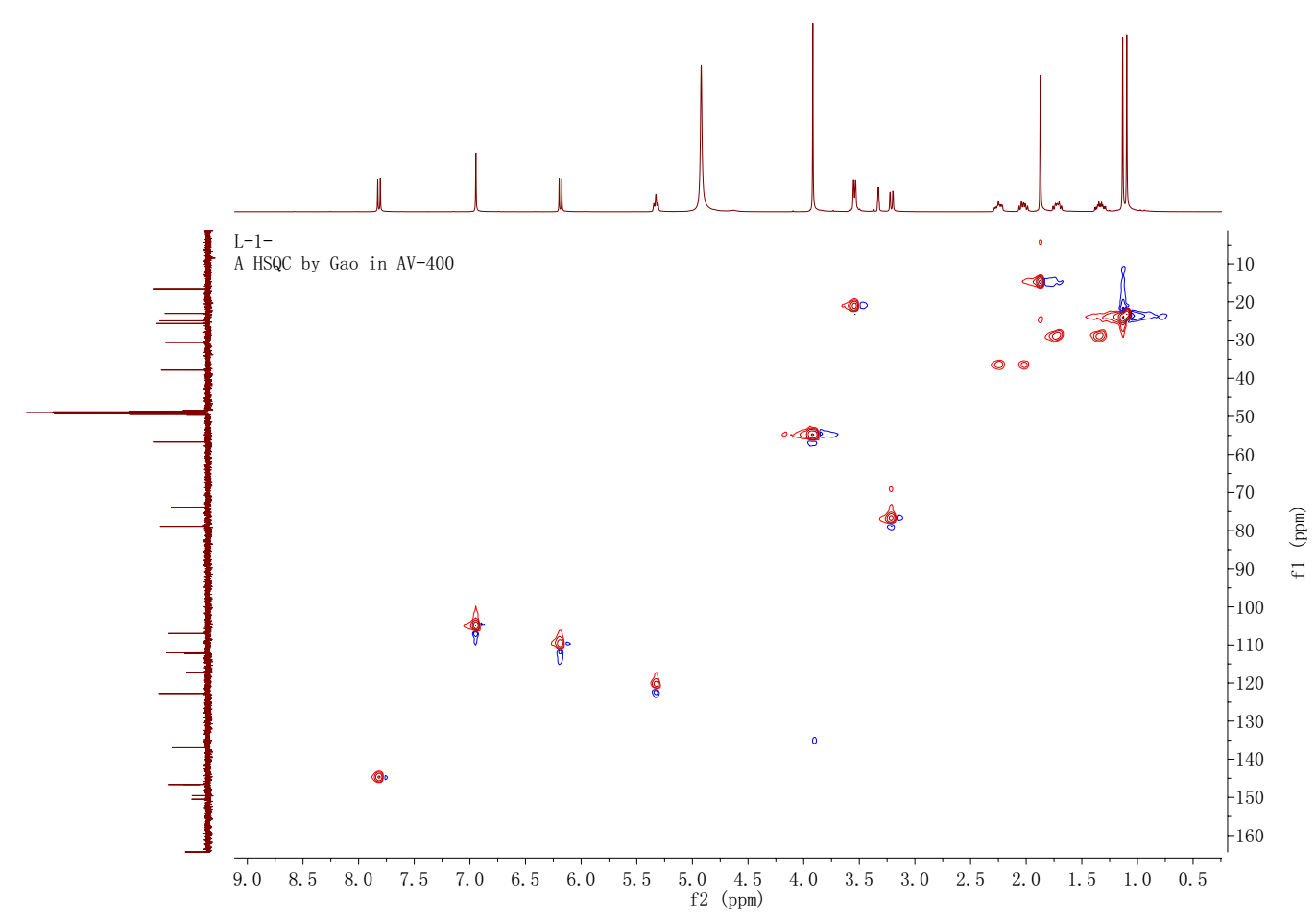


**Figure 26.** HSQC Spectrum of Compound 8

References

1. Liu, R.; Sun, Q.; Shi, Y.; Kong, L., Isolation and purification of coumarin compounds from the root of Peucedanum decursivum (Miq.) Maxim by high-speed counter-current chromatography. *J Chromatogr A* **2005,** 1076, (1-2), 127-32.

2. SANO, K.; YOSIOKA, I.; KITAGAWA, I., Studies on Coumarins from the Root of Angelica decursiva FR. et SAV. : II. Stereostructures of Decursin, Decursidin, and Other New Pyranocoumarin Derivatives. *Chemical and Pharmaceutical Bulletin* **1975,** 23, (0009-2363), 20-28.

3. Kuo; Yueh-Hsiung; Lin, S.-L., Chemical components of the whole herb of Mosla dianthera. *Chemical & Pharmaceutical Bulletin* **1999,** 47, (8), 1152-1153.

4. Liu, R.; Sun, Q.; Sun, A.; Cui, J., Isolation and purification of coumarin compounds from Cortex fraxinus by high-speed counter-current chromatography. *J Chromatogr A* **2005,** 1072, (2), 195-9.

5. Lee, D. Y.; Song, M. C.; Yoo, K. H.; Bang, M. H.; Chung, I. S.; Kim, S. H.; Kim, D. K.; Kwon, B. M.; Jeong, T. S.; Park, M. H.; Baek, N. I., Lignans from the fruits of Cornus kousa Burg. and their cytotoxic effects on human cancer cell lines. *Arch Pharm Res* **2007,** 30, (4), 402-7.
