# Supplementary material for: Endothelium-Independent Vasodilatory Effects of Isodillapiolglycol Isolated from Ostericum citriodorum
Source: Molecules. 2020 Feb 17;25(4):885. doi: 10.3390/molecules25040885 (PMC7070945; doi:10.3390/molecules25040885)
Supplement: Supplementary file 1 [file molecules-25-00885-s001.zip › Supplementary Materials for vasodilatory effects of isodillapiolglycol.docx]

The vasorelaxant effects of isodillapiolglycol (IDG) in aortic rings pre-contracted with NE (3 mM) or KCl (60 mM) at different times (10,20,30 min). The results showed that the effect was not statistically significant at different time points, suggested that IDG can reach the relaxation plateau after incubation for 10 min.


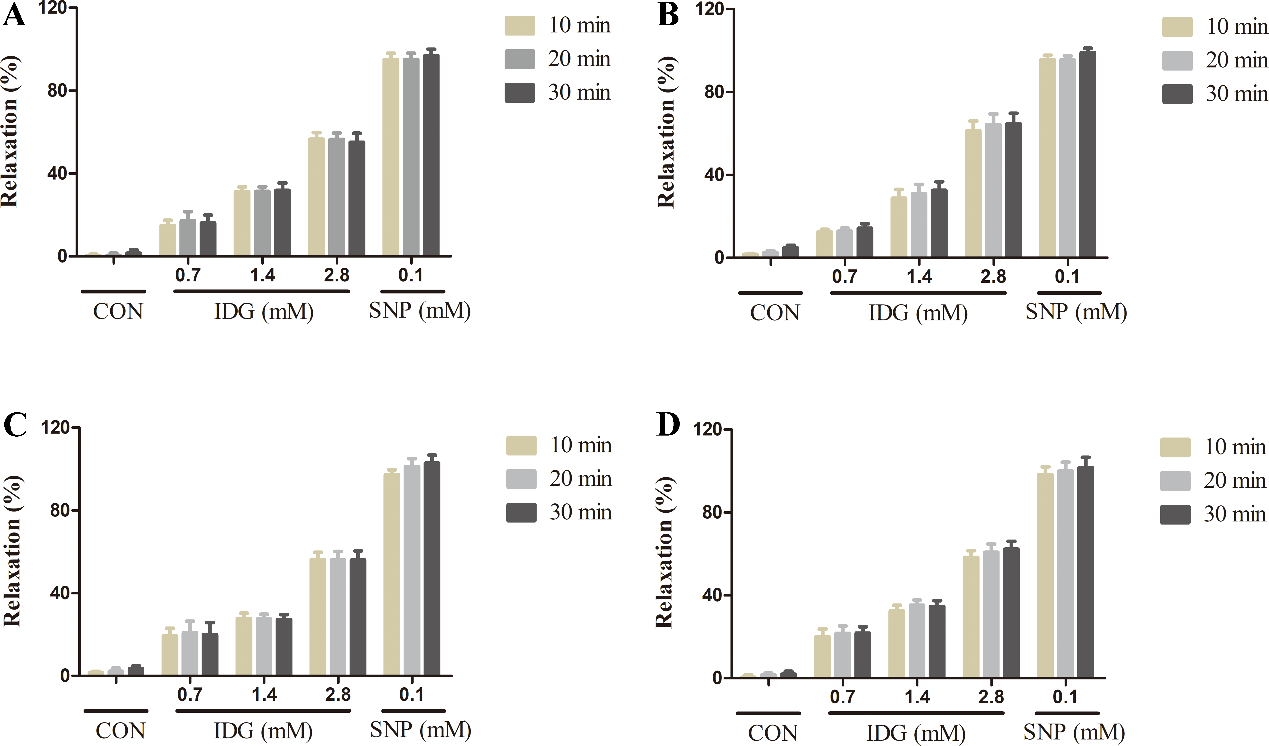


**Figure 1.** IDG directly caused relaxation in the vascular smooth muscle cells of aorta rings at different times. Endothelium-(**A**) intact or -(**B**) denuded aorta rings pre-contracted with NE (3 mM), Endothelium-(**C**) intact or -(**D**) denuded aorta rings pre-contracted potassium chloride (KCl) (60 mM). Values are means ± SEM (*n* = 6). Relaxation for IDG at 10 min was compared with relaxattion after pre-incubation for 20, 30 min using two-way ANOVA. **p* < 0.05 vs. 20 min group. ^#^*p* < 0.05 vs. 30 min group.
