# Supplementary material for: Endothelium-Independent Vasodilatory Effects of Isodillapiolglycol Isolated from Ostericum citriodorum
Source: Molecules. 2020 Feb 17;25(4):885. doi: 10.3390/molecules25040885 (PMC7070945; doi:10.3390/molecules25040885)
Supplement: Supplementary file 1 [file molecules-25-00885-s001.zip › Response to Reviewers.docx]

Dear reviewers,

Thanks very much for your hard work and kind help trying to improve our manuscript. We’ve studied your comments carefully and made corresponding changes in the paper. Please find detailed revisions as below:

Reviewer #1:

1) To understand why *O. citriodorum* is used for the treatment of AP, coronary artery disease, and hypertension as herbal medicine, please add more information on *O. citriodorum* in the Introduction (p. 2, lines 45−48). This information will help the readers to understand the importance of the present study.

**Answer:** *Ostericum citriodorum* is a traditional Chinese herb for treatment of angina pectoris, stomach pain and abdominal pain, its effect was clearly recorded in C*hina's Handbook of Common Chinese Herbal Medicine (1969)*, *National Compendium of Chinese Herbal Medicine (1996)*, *Cihai of Traditional Chinese Medicine (1998)*, *Chinese Materia Medica dictionary (2006)*, etc. In modern clinical research, "Huxinxin Capsule" with *O. citriodorum* as the main medicine treats angina and coronary heart disease.

2) The authors need to consider whether IDG can be absorbed from intestine and act in *in vivo* condition.

**Answer:** In this article, we mainly reported the vasodilatory effect of isodillapiolglycol in vitro, and the way of absorption and function of isodillapiolglycol in vivo will be developed and reported in subsequent experiments.

3) The authors should pay more attention while drawing the scheme. In Fig. 5B and 7, the authors explained that activation of Ca^2+^-dependent K^+^ channels induces Ca^2+^ influx into the cells, although it causes K^+^ efflux out of the cells resulting in hyperpolarization. Besides, since these ideas are included in the scheme (Fig. 7), Fig. 4B and 5B are not needed.

**Answer:** We have modified the content of the article based on your comments

4) The authors should analyze the dose-dependency of IDG-induced vasorelaxation before describing it in the text (Abstract, Results, and Discussion sections).

**Answer:** We have modified the content according to your suggestions, dose-dependent vasodilation effect of IDG is first analyzed in the summary as well as in the results and discussion

5) The authors should add information on the concentrations of the antagonists (VER, TEA, and BaCl_2_) used in the present study to the Methods (p. 9, sections 4.8, and 4.9).

**Answer:** It was our negligence without indicating the concentration information of the antagonists. The concentrations of these antagonists used in the experiments are: VER (10 μM), TEA (1 mM), BaCl_2_ (10 μM). Simultaneously, we have added this information to the article based on your suggestions.

6) In the Methods section, the authors stated that IDG was added for 30 min to determine its vasorelaxant effects (p. 8, line 237). Is such quite a long duration needed to reach the relaxation plateau? Can the authors display the data chart of the relaxation in the text? Is a 10-min treatment with IDG sufficient for determining the effects of IDG on extracellular Ca^2+^ influx (p. 9, line 247)?

**Answer:** In our experiment, the aortic rings tension which were contracted with NE were tested after IDG incubation for 10、20、30min. The results showed that the vasorelaxant effect of IDG was not statistically significant at different time points, suggested that IDG can reach the relaxation plateau after incubation for 10 min. Therefore, in the study of determining the effects of IDG on extracellular Ca^2+^ influx, we treatment the aortic rings with IDG for 10min. We did not express this result explicitly, the data in the article has been adjusted, the data listed in the article is the change in vascular tone after pre-incubation with the IDG for 10 minutes. At the same time, the aortic rings tension measured after IDG incubation for 10, 20, 30 minutes is listed in the attachment.

7) Generally, NE-induced contraction is caused by extracellular Ca^2+^ influx through not only ROCC but also VDCC together with Ca^2+^ release from the SR. This is supported by the fact that VDCC inhibitors, i.e., nifedipine, amlodipine, etc., can inhibit vasoconstriction, resulting in the reduction of blood pressure (that is the main reason for the use of these drugs as antihypertensive agents). Did NE induce adequate contraction to determine IDG-induced relaxation in the presence of verapamil (Fig. 4)?

**Answer:** As you mentioned, the presence of verapamil (VER, a L-type Ca^2+^ channel blocker) affected the changes of aortic rings tension contracted with NE. When the maximum contraction of the control group caused by NE was recorded as 100%, the maximum contractile tension of the vascular ring pre-incubation with VER was 74.2%. After IDG was added later, we can observe that both groups of aortic rings have a certain degree of relaxation, but the pre-incubated group with VER is significantly lower than the control group. Here we can draw two conclusions. First, the aortic rings incubated with IDG will still relaxation when it was pretreated with VER, indicating that IDG can still exert vasodilatory effect when the L-type Ca^2+^ channel was blocked; this vasodilatory effect was lower than the control group, indicating that the blocking of L-type Ca^2+^ channel affects the vasodilatory effect of IDG. Therefore, through this experiment, it can be shown that the vasodilatory effect of IDG is not only related to the inhibition of external Ca^2+^ influx through L-type Ca^2+^ channel, but also to the inhibition of internal Ca^2+^ release.

8) In Fig. 6, intracellular Ca^2+^ level in the VSMCs increased following the addition of CaCl_2_ alone in the presence of Ca^2+^-free solution to a similar level to that of KCl. Please explain what mechanisms were responsible for such findings.

**Answer:** I’m sorry that we didn't clearly express our thoughts in this experiment.

The experiment was divided into two groups, one of group was to incubate the vascular smooth muscle cells (VSMCs) with the IDG for 10 min in Ca^2+^-free Krebs’, and then add NE or CaCl_2_ instead of adding CaCl_2_ alone. NE can cause intracellular Ca^2+^ release and extracellular Ca^2+^ influx, therefore, when NE stimulates the VSMCs, the Ca^2+^ fluorescence intensity of the IDG groups was significantly lower than the control group, means that the addition of the IDG inhibits the release of internal Ca^2+^, subsequently, the addition of CaCl_2_ (2.5 mM) is equivalent to providing an external Ca^2+^ environment that caused the fluorescence increase of [Ca^2+^]_in_ in each group, similarly, the fluorescence intensity of the IDG groups was significantly lower than that of the control group, means that IDG can also inhibit external Ca^2+^ influx.

In another group, the cells were incubated with the IDG for 10 minutes in Krebs’ before KCl was added. Pre-incubation of KCl will caused extracellular Ca^2+^ influx in Krebs’, since the Krebs’ solution contains 1.6 mM CaCl_2_, therefore, the fluorescence intensity of this group looks similar to that in the previous experiment after adding CaCl_2_ (2.5 mM) to the Ca^2+^-free Krebs’.

9) The authors mentioned L-type Ca^2+^ channels (LTCC, Results 2.4, p. 3) and voltage-dependent Ca^2+^ channels (VDCC, Discussion, Fig. 7) in the text. Did they consider that these are two different types of Ca^2+^ channels?

**Answer:** Voltage-dependent Ca^2+^ channels include three subtypes of L, T, N. Among them, L-type Ca^2+^ channel have a long opening time and slow inactivation, which is the main pathway for extracellular Ca^2+^ influx.

Reviewer #2:

**ABSTRACT**

1. Abstract is a not a good summary of the work performed.

Line 17-20: English should be revised, sentences does not match.

However, some abbreviations appear without a previous meaning (Ex SD= Sprague Dawley).

Line 17-20: I suppose the authors want to say that “IDG inhibits CaCl2 induced contraction”.

**Answer:** We have modified the article based on your comments and adopted English editing services.

**INTRODUCTION**

2. English should be revised to correct some expressions along this section (line 43, line 44…)

Line 46-47: Authors indicate in this section that “Ostericum citriodorum is an herbal medicine used to treat AP”, however the reference to scientifically support this affirmation is an article on rat thoracic aorta.

**Answer:** It is our negligence that did not elaborate the basis of *Ostericum citriodorum* for treating angina pectoris (AP). As a plant with a native range in China, *O. citriodorum* is widely used to treat AP, coronary disease and hypertension. It is clearly recorded in *China's Handbook of Common Chinese Herbal Medicine*, *National Compendium of Chinese Herbal Medicine*, and *Cihai of Traditional Chinese Medicine*. In modern clinical research, “Huxin Capsule” with was composed of *O. citriodorum* has a good effect for treating AP in clinical practice. We have supplemented this part of the article with your comments based on your comments.

3. Lines 51-53: The authors mention another active principle of *Ostericum citriodorum*, the isoapiole. It is not clear if isoapiole and IDG are related molecules concerning structure.

**Answer:** Isoapiole ( Figure 1A) was separated from petroleum ether extract of *Ostericum citriodorum*, isodillapiolglycol (IDG) (Figure 1B) was separated from ethyl acetate extract of *O.citriodorum* , both of them are phenylpropanoids.

Figure 1. Chemical structure of isoapiole **(A)** or isodillapiolglycol **(B)**.

**RESULTS**

- General: the authors measured concentrations of IDG in mcg/ml. I think that it will be better to measure in molar units, once is a molecule with identified structure.

**Answer:** Thank you for pointing this out, we have modified it according to your comments.

4. - Line 57-59: The authors does not mention IDG.

**Answer:** Based on your suggestions, we have modified this part accordingly. In this part, we identified the compound as an IDG by structural identification, so we first describe its structure, and finally compare the hydrogen spectrum data with the literature to determine that this compound is IDG.

5. Figure 2 has bad quality. We can barely read the drugs names and labels. Figure A must contain somewhere that this is the effect on NE contraction. Same thing for figure B and KCl. Why the authors did not comment the SNP effects? The authors did not indicate the test that they used to perform statistical analysis.

**Answer:** We have modified the pictures according to your requirements. On the other hand, we also perform statistical analysis on the efficacy of IDG and SNP.

6. - Line 79-80: authors claim that it is not significantly different. What they did compare? Effects or EC_50_? Which test they used? Why they did not compare effect on NE and effect on KCl contractions? These contractions are induced by different mechanisms.

**Answer:** We study the vasodilatory effects of IDG contracted with NE or KCL in endothelium-intact and the endothelium-denuded aortic rings, and compared their differences by calculating EC_50_ values.

NE stimulates vasoconstriction by activating the receptor-operated Ca^2+^ channel (ROCC) to promote extracellular Ca^2+^ influx and release of intracellular Ca^2+^, while KCl is by activating the voltage-dependent Ca^2+^ channel (VDCC) to promote extracellular Ca^2+^ influx to cause vasoconstriction. These contractions are induced by different mechanisms, but both act by Ca^2+^ channels cause vasodilation. Through this experiment, we proved that IDG can play the vasodilation effect by intervening Ca^2+^ channels, but we did not further analyze whether the intervention effect of the two Ca^2+^ channels is the same. We will further analyze this in subsequent experiments.

7. Figure 3 has bad quality. The authors say that they show the relaxant effect of DPG, but this is not true (“y axis” is contraction and “x axis” is CaCl_2_ concentration). I think that this figure show the effect of pre-incubation with DPG on…, which is an inhibitory effect. The authors say in line 88: “IDG (200, 400, 800 μg/mL) significantly inhibited the concentration-response contraction of CaCl2”. The x scale in this figure must be logarithmic to better show the effects. The authors did not indicate the test that they used to perform statistical analysis.

**Answer:** We have modified the pictures and content in the article based on your comments.

8. Line 89-90: Sentence must be changed, it is confusing in actual form.

**Answer:** We have modified this section and adopted MDPI English Editing Services

9. Lines 86-90: the authors does not describe all the effects shown in figure 3. For example, the significant differences at the different concentrations. Why the authors did not make EC_50_ parameters to compare them?

**Answer:** We have re-described the effect reflected in the figure according to your suggestion. Here we only compared the vasodilatory effect of IDG at different CaCl_2_ concentrations, we want to through this experiment proved that IDG can inhibit vasoconstriction which was caused by external Ca^2+^ influx.

10. Figure 4 has bad quality. A big question here is how the author calculated the percent of relaxation by IDG in “control” and “verapamil” cases. Because contractility by NE in presence or absence of verapamil are very different in intensity. These effects can induce a misrepresentative analysis when to study the IDG effect. The authors did not indicate the test that they used to perform statistic analysis.

**Answer:** After noradrenaline (NE) stimulation of vascular rings with or without verapamil (VER), it can be observed that there is a difference contractile tension between the two groups, when the maximum contraction of the control group caused by NE was recorded as 100% , the maximum contractile tension of the vascular ring pre-incubation with VER was 74.2%. With IDG added later, it had a vasorelaxant effect of both groups, but the degree of relaxation was different. In the presence or absence of VER, the maximal vasorelaxant effects were 24.51 ± 2.22% and 63.85 ± 6.09%, respectively. It was suggested that IDG still has vasodilating effect when L-type Ca^2+^ channel was blocked, on the other hand, this vasorelaxant effect was significantly lower than the control group without VER, means that the vasodilatation of IDG was blocked by VER.

We have modified the content of the article based on your comments and fully described the statistical analysis.

11. Line 97-102: This paragraph must be completely changed to adequately describe the effects.

**Answer:** We have re-described this part of the experiment and adopted MDPI English editing services.

12. Figure 5 has bad quality. The legend must be completely changed. Results A and figure concerning mechanism (B) must be in different figures. The authors did not indicate the test that they used to perform statistical analysis.

**Answer:** We have modified the graph accordingly and described the statistical analysis in detail.

13. Lines 119-127: English should be deeply revised. This paragraph do not explain the observed effects in a comprehensive manner.

**Answer:** We have modified this paragraph and adopted MDPI English Editing Services.

We first incubated the vascular smooth muscle cells (VSMCs) with the IDG for 10 min, then added NE or CaCl_2_ in order to detect the change in intracellular fluorescence density. NE can cause intracellular Ca^2+^ release and extracellular Ca^2+^ influx, which is manifested as an increase in the fluorescence intensity of the VSMCs. Therefore, we incubated the VSMCs in a Ca^2+^-free Krebs’, When NE stimulates the vascular ring, The fluorescence intensity of the IDG group was significantly lower than that of the control group, means that the addition of the IDG inhibits the release of internal Ca^2+^, subsequently, the addition of CaCl_2_ (2.5 mM) is equivalent to providing an external Ca^2+^ environment that caused the fluorescence increase of [Ca^2+^]in in each group, similarly, the fluorescence intensity of the administration group was significantly lower than that of the control group.

14. Figure 6 has bad quality. In this case, this is more crucial to analyze the pictures shown and confirm the observed effects represented in the part B. The authors did not indicate the test that they used to perform statistical analysis in the legend.

**Answer:** We have modified the pictures according to your requirements and pointed out the statistical analysis in detail

**DISCUSSION**

15. The authors focused here, and in the introduction, on angina pectoris (AP), however the effect artery used here is rat aorta, which is not a myocardial artery. I this is not corresponding with the data obtained, which will be the case if they used a rat myocardial artery, for example.

**Answer:** The question you asked is of great significance to our experiment. As we mentioned in the introduction, there are many ways to treat angina pectoris. One of the important methods is to dilate blood vessels with drugs. Therefore, the focus of our experiment is to investigate the vasodilating effect of IDG。At the same time, the coronary arteries and thoracic aorta are structurally similar. We verified that IDG can inhibit cell contraction caused by calcium influx and internal calcium release in VSMCs by using VSMCs. Therefore, it can be proved that the vasodilation effect of IDG Regulated by calcium channels.

On the other hand, when reviewing relevant literature and studying the vasodilation effect of drugs [1-3], the isolated vascular rings used are mostly thoracic aortic rings, and the coronary arteries are thin, which is easy to cause damage when the endothelium is stripped. Therefore, when we are not sure whether IDG has a vasodilating effect or how this effect is generated, we use the more commonly and recognized thoracic aortic ring for experiments. And we will use the coronary vascular ring for related experimental research in subsequent studies.

1.	Jo, C.; Kim, B.; Lee, S.; Ham, I.; Lee, K.; Choi, H. Y., Vasorelaxant Effect of Prunus mume (Siebold) Siebold & Zucc. Branch through the Endothelium-Dependent Pathway. *Molecules* **2019,** 24, (18).

2.	Kim, B.; Lee, K.; Chinannai, K. S.; Ham, I.; Bu, Y.; Kim, H.; Choi, H. Y., Endothelium-Independent Vasorelaxant Effect of Ligusticum jeholense Root and Rhizoma on Rat Thoracic Aorta. *Molecules* **2015,** 20, (6), 10721-33.

3.	Senejoux, F.; Girard, C.; Kerram, P.; Aisa, H. A.; Berthelot, A.; Bevalot, F.; Demougeot, C., Mechanisms of vasorelaxation induced by Ziziphora clinopodioides Lam. (Lamiaceae) extract in rat thoracic aorta. *J Ethnopharmacol* **2010,** 132, (1), 268-73.

16. Discussion contains descriptions that must be in the introduction (VDCC, ROCC?).208-215

**Answer:** We have made changes to this section based on your suggestions.

17. Line 152-163: The authors did not demonstrate, with the results shown, any action of IDG on these channels. This affirmation is circumstantial, IDG can reduce Ca^2+^ acting in another mechanism and this effect can be a consequence.

**Answer:** The idea of our experiment is to investigate whether IDG has a vasodilating effect, and to explore how this vasodilating effect occurs. We used NE or KCl to induce vasoconstriction. NE promotes extracellular Ca^2+^ influx and release of Ca^2+^ by activating ROCC channels on the cell membrane, while KCl promotes extracellular Ca^2+^ influx by activating VDCC channels in cells. Regardless of NE or KCl, both of them are increase the intracellular Ca^2+^ concentration by activating Ca^2+^ channels on the cell membrane, which leads to vasoconstriction. We have demonstrated that IDG can relax vasoconstriction caused by KCL and NE in vitro experiments. Cell experiments have shown that IDG can inhibit the increase of intracellular Ca^2+^ concentration caused by KCl or NE. These experiments have shown that IDG's vasodilating effect is related to Ca^2+^ channels, which can inhibit intracellular Ca^2+^ release and external Ca^2+^ influx. But as you said, we have verified that this vasodilatory effect is related to VDCC and ROCC. There is no analysis on how it acts on VDCC and ROCC to inhibit internal Ca^2+^ release and external Ca^2+^ influx, but this is exactly what we are doing and we will report on this in subsequent experiments.

18. Lines 164-168: here the authors say that IDG acts by activating K_Ca_ channels (??).

**Answer:** Activation of potassium channels both in the vascular smooth muscle and endothelium causes hyperpolarization which leads to vasorelaxation. We used the inhibitors TEA (5 mM) to block Ca^2+^-activated K^+^ (K_Ca_) channels. IDG induced relaxation was partially decreased due to pretreatment with TEA. These data indicate the activation of K_Ca_ channels, may be involved in IDG-induced vasorelaxation.

19. Figure 7 shows that IDG acts in VDCC, ROCC, K_Ca_ channels and also on exit of Ca^2+^ from endoplasmic reticulum. In my opinion, these are only possibilities or hypothesis, because the results shown does not demonstrate that these all are targets of IDG.

**Answer:** We all know that NE can promote extracellular Ca^2+^ influx and release of Ca^2+^ by activating ROCC channels on the cell membrane, and KCl can activate VDCC on the membrane and promote extracellular Ca^2+^ influx. In vitro experiments, IDG can relax vasoconstriction caused by KCl or NE, indicating that the vasodilatory effect of IDG was related to VDCC or ROCC. Further through cell experiments, we observed that the presence of IDG may inhibit the increase of intracellular Ca^2+^ concentration in vascular smooth muscle cells through VDCC or ROCC channels. On the other hand, in vitro experiment, when the aortic rings were incubated with TEA, a blocker of the Ca^2+^-activated K^+^ channel, we can observe that the vasodilation effect of IDG decreased significantly, which indicate the vasodilatory effect of IDG may relate to activation of Ca^2+^-activated K+ channel.

Through this experiment we proved that vasodilation effect of IDG is related to VDCC, ROCC, or Ca^2+^-activated K^+^ channel, but further research is needed to study how the IDG regulates intracellular Ca^2+^ concentration through these channels to relax the aortic rings. These will be further researched in our subsequent experiments, thank you very much for your comments.

**MATHERIAL & METHODS**

20. - Line 185: SD is an abbreviation without meaning.

**Answer:** I'm sorry it was our negligence that caused this typographical error and we have modified it

21.- A7r5 cells are in the methods but tgeir use is not mentioned in the results. Culture procedures of these cells are not mentioned

**Answer:** We haven't described it clearly here, which will cause you inconvenience. A7r5 is a rat thoracic aortic smooth muscle cell, and we have used this cell in the observation of IDG on cell experiments. At the same time, according to your opinion, we intend to supplement the culture procedures for A7r5 cells.

22. - Statistical analysis have different pos hoc tests that are not indicated in the results section.

**Answer:** We have supplemented and improved the statistical analysis based on your suggestions.
